# Variabilities in retinal function and structure in a canine model of cone-rod dystrophy associated with *RPGRIP1* support multigenic etiology

**DOI:** 10.1038/s41598-017-13112-w

**Published:** 2017-10-09

**Authors:** Rueben G. Das, Felipe Pompeo Marinho, Simone Iwabe, Evelyn Santana, Kendra Sierra McDaid, Gustavo D. Aguirre, Keiko Miyadera

**Affiliations:** School of Veterinary Medicine, University of Pennsylvania, Philadelphia, PA 19104 USA

## Abstract

Defects in the cilia gene *RPGRIP1* cause Leber congenital amaurosis and cone-rod dystrophy in humans. A form of canine cone-rod dystrophy (cord1) was originally associated with a homozygous insertion in *RPGRIP1* (*RPGRIP1* ^ins/ins^) as the primary disease locus while a homozygous deletion in *MAP9* (*MAP9* ^del/del^) was later identified as a modifier associated with the early onset form. However, we find further variability in cone electroretinograms (ERGs) ranging from normal to absent in an extended *RPGRIP1* ^ins/ins^ canine colony, irrespective of the *MAP9* genotype. Ophthalmoscopically, cone ERG^absent^ *RPGRIP1* ^ins/ins^ eyes show discolouration of the tapetal fundus with varying onset and disease progression, while sd-OCT reveals atrophic changes. Despite marked changes in cone ERG and retinal morphology, photopic vision-guided behaviour is comparable between normal and cone ERG^absent^ *RPGRIP1* ^ins/ins^ littermates. Cone morphology of the dogs lacking cone ERG are truncated with shortened outer and inner segments. Immunohistochemically, cone ERG^absent^ *RPGRIP1* ^ins/ins^ retinas have extensive L/M-opsin mislocalization, lack CNGB3 labelling in the L/M-cones, and lack GC1 in all cones. Our results indicate that cord1 is a multigenic disease in which mutations in neither *RPGRIP1* nor *MAP9* alone lead to visual deficits, and additional gene(s) contribute to cone-specific functional and morphologic defects.

## Introduction

The photic environment has played a significant role in retinal evolution in animals, as the size of the retina and the density and population of photoreceptors (rods and cones) adapt to changes in habitat illumination^1^. Cone photoreceptors aid in photopic (bright light) and colour vision and promote visual acuity while rods primarily function in scotopic (dim light) condition. Although cones constitute only 3–5% of the total photoreceptors in the human retina, they are used more frequently than rods, as we are less likely to be exposed to the dark in the modern world. Therefore, cone-rod dystrophies (CRDs), a form of inherited degenerative retinal disorders that result in an initial loss of cones and in which rod impairment is either delayed or reduced, are particularly devastating.

Photoreceptors consist of an inner segment (IS) and an outer segment (OS) that are connected by a narrow photoreceptor sensory cilium. Proper functioning of photoreceptors requires continual transport of proteins, lipids, and metabolites from the IS to OS through the cilia^2^, followed by rapid renewal of the OS as it is subsequently shed and phagocytized by the retinal pigment epithelium (RPE). Hence, disruption of cone-derived, photopic vision can be caused by defects in cone structure and/or function, including alterations in the transport of key macromolecules across the cones.

The cone-predominant nature of CRDs manifests as decreased central visual acuity and defects in colour vision. With further disease progression, dysfunction involves the rods, eventually leading to blindness^3^. Cone-rod dystrophy 1 (cord1) is a canine form of CRD previously described in a miniature long-haired dachshund (MLHD) research colony where the disease segregated autosomal recessively^4^. Affected pups had fundus abnormalities, such as granular tapetum that developed retinal thinning and vascular attenuation by 25 weeks of age. The cone-derived electroretinogram (ERG) was greatly reduced at this stage, while the rod-derived ERG remained normal, despite signs of degeneration. By 40 weeks, the retina was severely degenerated, with undetectable cone- or rod-derived ERGs^5^. This research colony was used to map a disease-specific 14 Mb interval^6^ that was further narrowed down to 1.74Mb^7^ on canine chromosome 15. Among the positional candidate genes, retinitis pigmentosa GTPase regulator-interacting protein 1 (*RPGRIP1*) was found to harbour a homozygous 44 bp insertion (*RPGRIP1* ^ins/ins^) in exon 2 (now revised to exon 3; CanFam3.1) that segregated with cord1 in the colony^6^. While the insertion was initially predicted to result in a frame shift and protein truncation^6^, it was later shown that exon skipping occurs, allowing the main functional domain RID (RPGR-interacting domain) to remain intact^8^.

Despite the apparent straightforward monogenic role for *RPGRIP1* for causation of cord1 in the MLDH research colony, a significant proportion of *RPGRIP1* ^ins/ins^ MLHDs outside of the original research colony did not develop early-onset blindness, but instead were affected at a much later age, or remained clinically normal throughout life^7^. Furthermore, dogs of other breeds such as English Springer spaniel, Labrador retriever and French bulldog also harbour the *RPGRIP1* insertion, but are clinically unaffected^7,8^. Based on the discordance between *RPGRIP1* genotype and clinical phenotype, a genome-wide association study was undertaken that identified a locus ~30 kb downstream of *RPGRIP1* on canine chromosome 15 that influenced the age of onset of severe retinal degeneration or clinical blindness^9^. Recently, targeted-sequencing of this locus identified a ~22 kb deletion spanning part of the gene encoding microtubule associated protein 9 (*MAP9)* and the neighbouring *MAP9* partial pseudogene^10^. This deletion leads to a fusion transcript *MAP9*_*EORD*_ containing several deleterious variants at the region corresponding to the 3′UTR of *MAP9*.

While mutations in *RPGRIP1* and *MAP9* have been linked to cord1, accurate prediction of disease onset is still challenging. To study the disease course in genotype-ascertained dogs, we developed an out crossed canine colony whose founders included an *RPGRIP1* ^ins/ins^ MLHD from the original research colony in the UK^8^. Interestingly, none of the *RPGRIP1* ^ins/ins^ progeny developed clinical blindness by up to age 8 years. Despite the lack of visual defects, there was a distinct and marked difference in photopic cone-derived ERG that ranged from normal to reduced (cone ERG^reduced^) or complete absence (cone ERG^absent^) in these dogs. To tease out the factor responsible for this spectrum of cone responses, we examined the retinal structure by ophthalmoscopy, spectral domain optical coherence tomography (sd-OCT) and/or immunolabelling, and retinal function by ERG and vision-guided behaviour in *RPGRIP1* ^ins/ins^ mutants. Herein we report our findings that support a multigenic basis for cord1.

## Results

### Normal rod-derived but variable cone-derived ERG in *RPGRIP1*^ins/ins^ animals

**Figure 1:**
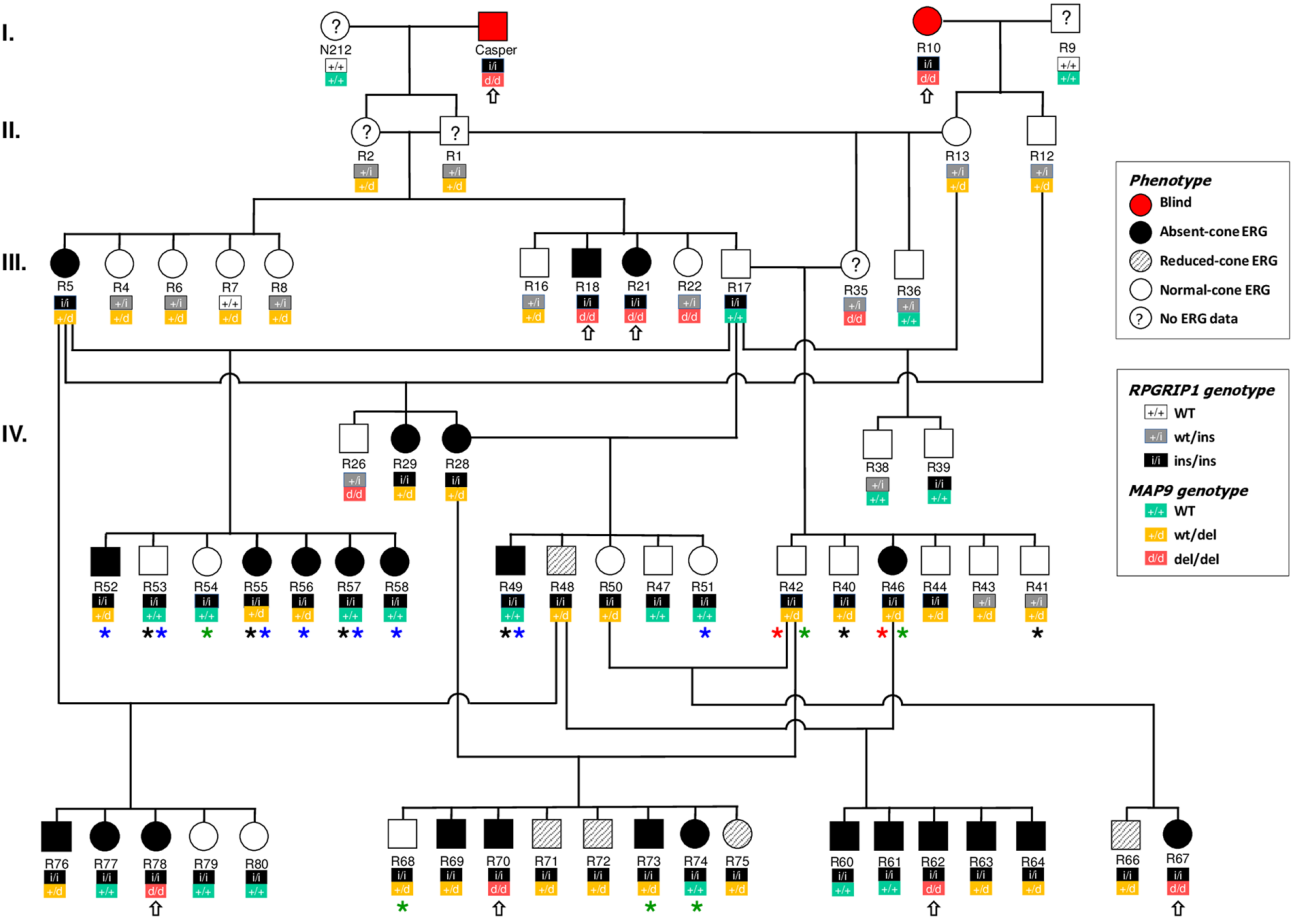
Segregation of *RPGRIP1* and *MAP9* mutations and phenotypic variability in the canine pedigree studied. Four unrelated dogs including three MLHDs (Casper, R9 and R10) founded the research colony. The symbols indicate the cone ERG status ranging from normal, reduced to absent, and clinical blindness. Below each symbol, the *RPGRIP1* (top) and *MAP9* (bottom) genotypes are shown where “i” and “d” represent the mutant alleles respectively. Animals that contributed to IHC, behaviour, qPCR, or Western blot studies are indicated with black, red, blue, or green asterisks respectively. Double homozygotes (*RPGRIP1* ^ins/ins^ *MAP9* ^del/del^) are indicated with open arrows. Square, male; circle, female.

**Figure 2:**
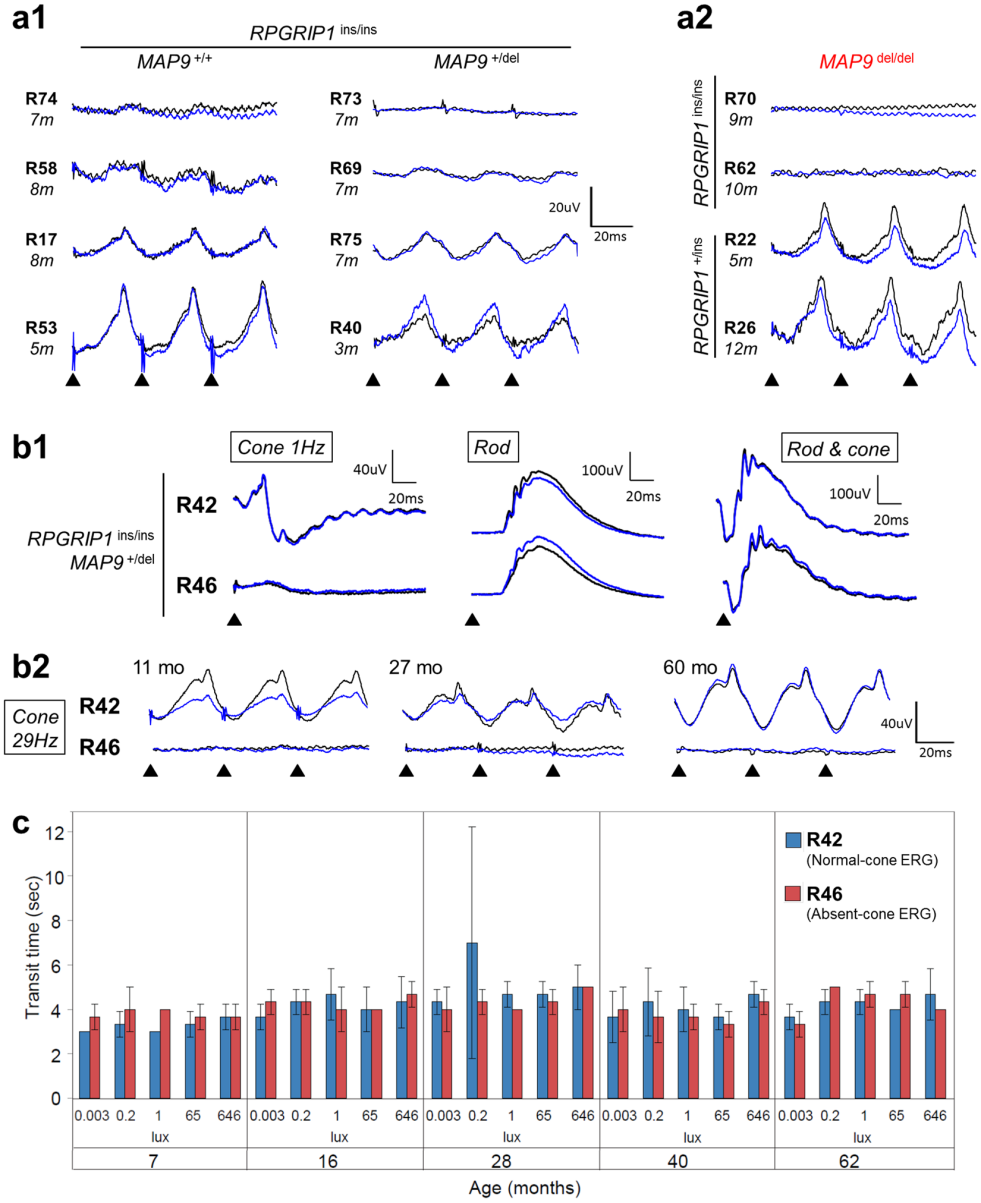
Representative ERG responses and vision-guided behaviour among *RPGRIP1* ^ins/ins^ animals. (**a,b**) ERG responses to light stimuli occurring at the time points indicated by bold triangles, for each right (black) and left (blue) eyes. (**a1**) Representative cone-ERG 29 Hz flicker responses of *RPGRIP1* ^ins/ins^ animals with either *MAP9* ^+/+^ or *MAP9* ^+/del^ are displayed in increasing order of ERG amplitude. The cone ERG phenotype ranges from absent to normal in both *MAP9* genotypic groups. (**a2**) Representative cone 29 Hz flicker ERG traces of *MAP9* ^del/del^ animals with and without concomitant *RPGRIP1* ^ins/ins^ mutation. All the double homozygotes (*RPGRIP1* ^ins/ins^ *MAP9* ^del/del^) in the colony were cone ERG^absent^. (**b1**) ERG traces of *RPGRIP1* ^ins/ins^ *MAP9* ^+/del^ littermates R42 and R46 at 5 yrs are shown. Single flash cone-derived ERG response was normal in R42 (“normal”) but diminished in R46 (“cone ERG^absent^”). Rod-derived and mixed ERG responses were comparable between the animals. (**b2**) Sequential cone-derived 29 Hz flicker responses are shown at 11, 27, and 60 mon where the normal (R42) and absent (R46) cone responses have remained stable over time. (**c**) Graphical representation of transit times (sec) in a vision-guided obstacle course in normal (R42) and cone ERG^absent^ (R46) animals under increasing illuminations (lux) are illustrated per observation time point. Visual function of these sibs has been shown previously at 6, 8 and 10 mon^8^ and therefore this is an extended study on both animals. Over the 5-year period, there was no difference in the transit time between the dogs at any of the light intensities. Each transit time shown is the average of three trials through an obstacle course under each illumination. Error bars represent ± SD.

The earliest sign of cord1 uniformly observed in the original research colony was the reduction of cone-derived ERG^5^. In a recent study, an age-at-onset modifier was identified in *MAP9* ^9,10^. To assess both rod and cone function in the current pedigree (Fig. 1), ERGs were recorded in *RPGRIP1* ^ins/ins^ animals as well as in *RPGRIP1* ^+/ins^ controls. Scotopic rod-derived ERG did not show any consistent or significant reduction across the different *RPGRIP1* genotypes. However, photopic cone-derived ERG (1 Hz and 29 Hz) varied significantly among *RPGRIP1* ^ins/ins^ animals, ranging from normal to reduced or completely absent (Fig. 2a). Extensive variation was observed within and across different litters, and this was independent of the *MAP9* genotype (Supplemental Table 3, Figs 1 and 2a). While the cone ERG^absent^ phenotype was seen among *RPGRIP1* ^ins/ins^ dogs of all *MAP9* genotypes, notably, all the double homozygotes (*RPGRIP1* ^ins/ins^ *MAP9* ^del/del^; n = 6) fell into the cone ERG^absent^ phenotype (Fig. 2a2). Those dogs that were homozygous for *MAP9* but not for *RPGRIP1* (i.e. *RPGRIP1* ^+/ins^ *MAP9* ^del/del^) had normal cone ERG (Fig. 2a2). There was no difference in rod-derived ERG responses across the *RPGRIP1* ^ins/ins^ dogs of all *MAP9* genotypes (Fig. 2b1). ERG findings remained largely consistent over time when repeated (Fig. 2b2), and none of the animals in the extreme phenotypic groups changed status to the other phenotypic extreme (e.g. from normal to cone ERG^absent^, and *vice versa*). Assessment of each L/M- and S-cone function in normal, cone ERG^reduced^, and cone ERG^absent^ animals showed that L/M- and S-cone ERGs were equally affected in the latter two groups, the cone ERGs of which were abnormal (Supplemental Fig. S1).

### Normal visual performance in cone ERG^absent^*RPGRIP1*^ins/ins^ animals

To assess both photopic and scotopic visual functions, two littermates (*RPGRIP1* ^ins/ins^ *MAP9* ^+/del^) that were either normal (R42) or cone ERG^absent^ (R46) were run through an obstacle avoidance course under different light intensity conditions periodically over a 5.5- year period (Fig. 2c; Supplemental Video 1). This pair had previously been studied at a younger age (6 through 10 months)^8^. Throughout the study period, the transit times remained comparable between the two animals. Furthermore, none of the 12 *RPGRIP1* ^ins/ins^ animals observed for over 2 years in the research colony developed clinically appreciable vision impairment or blindness up to ages 7.3 (normal) and 8 (cone ERG^absent^) years.

### Normal retinal structure at early age but progressive degenerative changes at later age in some *RPGRIP1*^ins/ins^ animals

**Figure 3:**
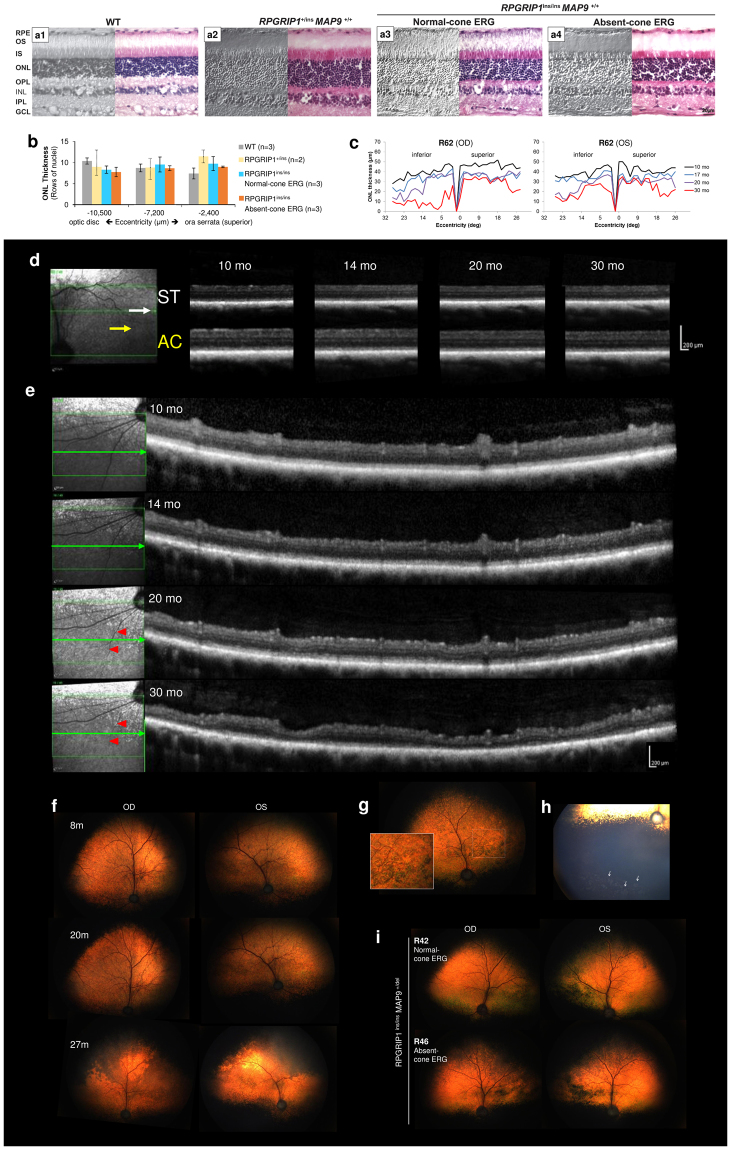
Normal retina in the first year with slow retinal degenerative changes observed at later age in some *RPGRIP1* ^ins/ins^ dogs. (**a**) Differential interference contrast (DIC) (left) and H&E-stained (right) images of WT, *RPGRIP1* ^+/ins^, and *RPGRIP1* ^ins/ins^ (normal or cone ERG^absent^) retinas at 9–11 mon were comparable without overt structural abnormality. RPE, retinal pigmented epithelium; OS, outer segment; IS, inner segment; ONL, outer nuclear layer; OPL, outer plexiform layer; INL, inner nuclear layer; IPL, inner plexiform layer; GCL, ganglion cell layer. (**b**) ONL thickness was quantitated by counting the rows of nuclei (/558μm length) at 2,400, 7,200, and 10,500 μm distances from the ora serrata. H&E-stained sections from WT (n = 3), *RPGRIP1* ^+/ins^ (n = 2), and *RPGRIP1* ^ins/ins^ (3 normal, 3 cone ERG^absent^) retinas at 9–11 mon were used. Error bars represent standard errors for groups of n = 3, and ranges (dotted line) in the group of n = 2. (**c**) Spider graphs of ONL thickness along the inferosuperior retina transversing the optic nerve, measured by manual segmentation from sd-OCT B-scans. Changes over time in each eye of a double homozygote *RPGRIP1* ^ins/ins^ *MAP9* ^del/del^ (R62; cone ERG^absent^) from 10 through 30 mon are shown. Progressive ONL thinning that is more pronounced in the inferior retina is observed in both eyes. (**d,e**) Changes in retinal/ONL thickness imaged by sd-OCT in the superotemporal (**d**, ST), area centralis (**e**, AC) and inferonasal (**e**) retinal areas in the double homozygote shown in (**c**). The left eye is shown and the arrows in the left cSLO image indicate the position of scanning. Progressive retinal/ONL thinning is observed in all retinal areas and is most pronounced in the inferonasal quadrant. Progressive vascular attenuation (arrow heads) was evident in the inferonasal scan (**e**). (**f**) Fundus changes over time in the double homozygote shown in (**c**–**e**). The tapetal fundus remained within normal limits until 20 mon. However, by 27 mon, dark discoloration had advanced from the superior periphery. Vascular attenuation and hyper-reflectivity were also noticed. (**g**) Fundus lesions in the right eye of an *RPGRIP1* ^ins/ins^ *MAP9* ^+/del^ dog (R48, cone ERG^severely reduced^) observed at 5 yrs. Multifocal circular lesions (magnified in inset) present across the tapetal fundus were condensed nasally. (**h**) Multifocal depigmentation in the far inferior non-tapetal fundus in the left eye of an *RPGRIP1* ^ins/ins^ *MAP9* ^+/del^ dog (R28, cone ERG^absent^) at 6 yrs. (**i**) Fundus of the littermate R42 and R46 shown in Fig. 2b,c, imaged at 5 yrs. R46 (cone ERG^absent^) showed initial signs of dark and mottling discoloration extending from the dorsal periphery while the R42 (normal cone-ERG) remained relatively normal.

To study the structure of the *RPGRIP1* ^ins/ins^ retina, histology was performed in young eyes, while extended *in vivo* observation were performed in other animals. *RPGRIP1* ^ins/ins^ retinas with the extreme phenotypes (i.e. normal or cone ERG^absent^) were used to increase the chance of identifying any changes. Retinal morphology studied by H&E staining was comparable across all the groups: wild type (WT) (n = 3; 2–5 yrs), *RPGRIP1* ^+/ins^ (n = 1; 9 mon), and *RPGRIP1* ^ins/ins^ that were normal (n = 2; 9–11 mon) or cone ERG^absent^ (n = 3; 9–11 mon) (Fig. 3a). There was no significant difference in ONL thickness in the superior and inferior retinal quadrants among the groups (Fig. 3b).

Although typical early retinal degenerative changes had been observed ophthalmoscopically in the *RPGRIP1* ^ins/ins^ founder animals (i.e. Casper, R10), all the subsequent *RPGRIP1* ^ins/ins^ progeny were found to have normal fundus in early life. However, with age, some cone ERG^absent^ mutants developed dark discoloration of the tapetal fundus, typically appearing as “mottled” dark areas extending from the superior tapetal-nontapetal junction (Fig. 3f). These occasionally coalescing darker regions were more extensive in the far superior fundus. Areas adjacent to the dark colouration had increased reflectivity from the underlying tapetum, indicating thinning of the retina. A variant of the fundus change in the form of irregular circular lesions against the dark discoloured background was also observed (Fig. 3g). In the non-tapetal fundus, multifocal patchy depigmentation was seen in some retinas (Fig. 3h). There was significant variability in the onset, progression, and appearance of the fundus lesions. While only a few *RPGRIP1* ^ins/ins^ animals have been followed into later age thus far, age-related progressive fundus changes were observed exclusively in the cone ERG^absent^ retinas. For example, when comparing a cone ERG^absent^ animal with its normal littermate harbouring identical genotypes (*RPGRIP1* ^ins/ins^ *MAP9* ^+/del^), the former was found to have developed more prominent dark superior discoloration by 5 years of age (Fig. 3i). While further longitudinal data are required to draw an association between the genotypes and fundus lesions, there was a tendency for the double homozygotes (*RPGRIP1* ^ins/ins^ *MAP9* ^del/del^) to have a more severe phenotype, with earlier onset and faster progression.

Fundus images captured by confocal scanning laser ophthalmoscope (cSLO) revealed changes comparable to those visualized by ophthalmoscope. Changes in the ONL thickness over time were determined by sd-OCT. In animals younger than 1 year, retinal structure and thickness were within normal limits. However, with age, sd-OCT revealed progressive reduction in ONL thickness among some of the cone ERG^absent^ mutants (Fig. 3c–e). These degenerative changes were observed in all quadrants of the retina but were most pronounced in the inferotemporal quadrant. Morphologic appearance in older subjects remains to be verified once tissues become available for analysis. Again, the double homozygotes (*RPGRIP1* ^ins/ins^ *MAP9* ^del/del^) had a more severe phenotype with rapidly progressing ONL thinning starting as early as 17 months of age compared to the *RPGRIP1* ^ins/ins^ *MAP9* ^+/del^ animals where ONL changes could not be visualized by sd-OCT until 4 years of age (Supplemental Table 3a). Notably, the one *RPGRIP1* ^ins/ins^ *MAP9* ^+/+^ animal (R17) with normal cone ERG has not shown ONL changes for up to 7 years of age. Together these data suggest that the additional defect in *MAP9* leads to a more severe phenotype among the *RPGRIP1* ^ins/ins^ animals.

### Abnormal cone photoreceptor structure in cone ERG^absent^*RPGRIP1*^ins/ins^ retina

**Figure 4:**
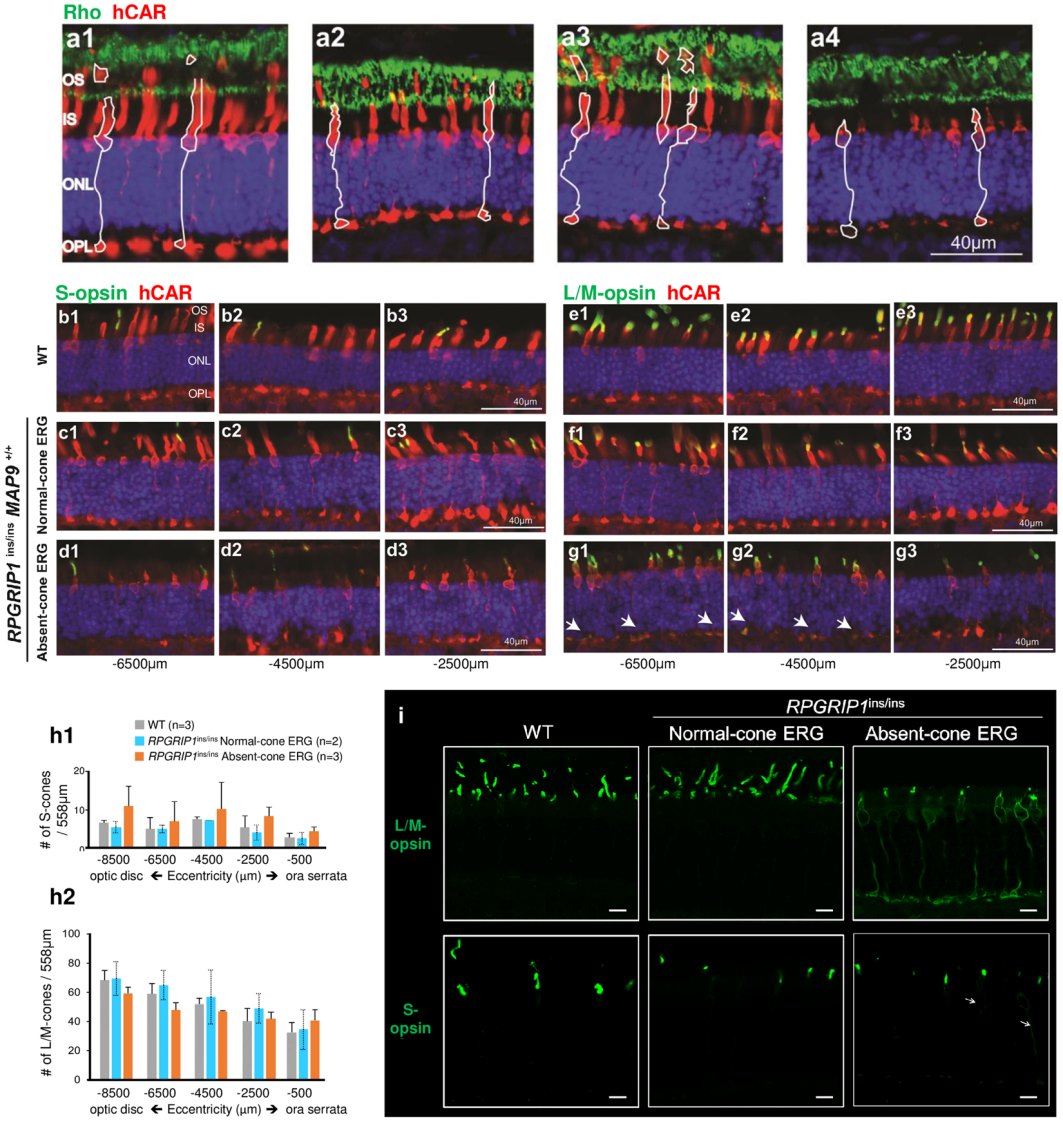
Abnormal cone photoreceptor structure in cone ERG^absent^ *RPGRIP1* ^ins/ins^ retina. (**a**) Double-immunolabelling of retinal cryosections with antibodies against rod opsin and human cone arrestin (hCAR) in WT (a1), *RPGRIP1* ^+/ins^ *MAP9* ^+/+^ (a2), and *RPGRIP1* ^ins/ins^ *MAP9* ^+/+^ animals that are normal (a3) or cone ERG^absent^ (a4). All samples had comparable rod structure with normal localization of rod opsin while the cone ERG^absent^ retina showed short cone OS and IS. The contours of representative cones have been outlined for each group. (**b**–**g**) Representative images of the retina double labelled with hCAR and S-opsin (**b**–**d**) or L/M-opsin (**e**–**g**) at 3 different positions relative to ora serrata in WT (b1–3, e1–3), normal (c1–3, f1–3) or con ERG^absent^ (d1–3, g1–3) *RPGRIP1* ^ins/ins^ retinas. White arrows indicate mislocalization of L/M-opsin in the cone ERG^absent^ retina which was observed at most of the retinal locations examined (g1–3). (**h**) Number of S- (h1) and L/M-cones (h2) per 558μm field in WT and normal or cone ERG^absent^ *RPGRIP1* ^ins/ins^ retinas. Each cone population was counted in the superior retinal quadrant starting −500 μm from the ora serrata towards the optic disc every 2,000μm. Error bars represent standard errors for groups of n = 3, and ranges (dotted line) for the group where n = 2. OS, outer segment; IS, inner segment; ONL, outer nuclear layer; OPL, outer plexiform layer. (**i**) Confocal microscopy images showing immunolabelling of WT and *RPGRIP1* ^in/ins^ (normal cone ERG and cone ERG^absent^) retinas with antibodies against L/M-opsin and S-opsin. Extensive opsin mislocalization to the cell body, axon, and synaptic pedicles were seen in the plasma membrane of L/M-cones, and rarely in S-cones (arrows) of the cone ERG^absent^ *RPGRIP1* ^ins/ins^ retina. Scale bar, 10 μm.

Due to the cone-specific nature of the ERG phenotype, cone photoreceptor structures were examined in normal and cone ERG^absent^ *RPGRIP1* ^ins/ins^ retinas. IHC using anti-human cone arrestin (hCAR) revealed a striking difference in the cone structure of cone ERG^absent^ retinas, where both the OS and IS were shortened (Fig. 4a4). While most cone OS were extremely small, some rare apparently normal cone OS were also observed. Cone OS that appeared fragmented and/or elongated most likely represented tissue processing artifacts. The overall structural differences in the cone ERG^absent^ *RPGRIP1* ^ins/ins^ retinas *vs* retinas of other phenotypic/genotypic groups were significant enough to allow easy distinction. Rod photoreceptors were normal with correct rod opsin localization in all the sample groups (Fig. 4a).

### Opsin mislocalization but no cone photoreceptor loss in the cone ERG^absent^*RPGRIP1*^ins/ins^ retina

To differentiate and characterize the two cone types, we examined the cone visual pigments (L/M- and S-opsins) that are normally expressed in the OS outer membrane of the respective cone types. Retinal cryosections of young (9–11 mon) *RPGRIP1* ^ins/ins^ animals and controls were used for IHC. Most strikingly, the OS of the cone ERG^absent^ *RPGRIP1* ^ins/ins^ retina were shorter, and as a result, had a more limited area of L/M-opsin immunolabelling consistent with the short OS structure; as well there was extensive L/M-opsin mislocalization to the plasma membranes of IS, cell body and synaptic pedicles (Fig. 4g,i). Consequently, the overall L/M-opsin labelling intensity in these retinas was not different from that of WT and cone ERG normal *RPGRIP1* ^ins/ins^ retinas, and only differed in distribution. S-opsin labelling pattern was comparable across the different sample groups, with only rare and subtle S-opsin mislocalization observed in the cone ERG^absent^ *RPGRIP1* ^ins/ins^ retina (Fig. 4d,i).

The number of L/M- and S-cone cells were then compared between normal and cone ERG^absent^ retinas. The density of each cone type was similar between the different genotypic and phenotypic groups (Fig. 4h). Lack of active cell loss was also confirmed by the absence of TUNEL positive cells in all retinal quadrants in the different sample groups. These findings indicate that despite the shortened cell morphology and opsin mislocalization, there is no significant loss in the cone cell population in the cone ERG^absent^ retinas before 1 year of age.

### Reduced or absent labelling of some photoreceptor OS proteins in the cone ERG^absent^*RPGRIP1*^ins/ins^ retina

**Figure 5:**
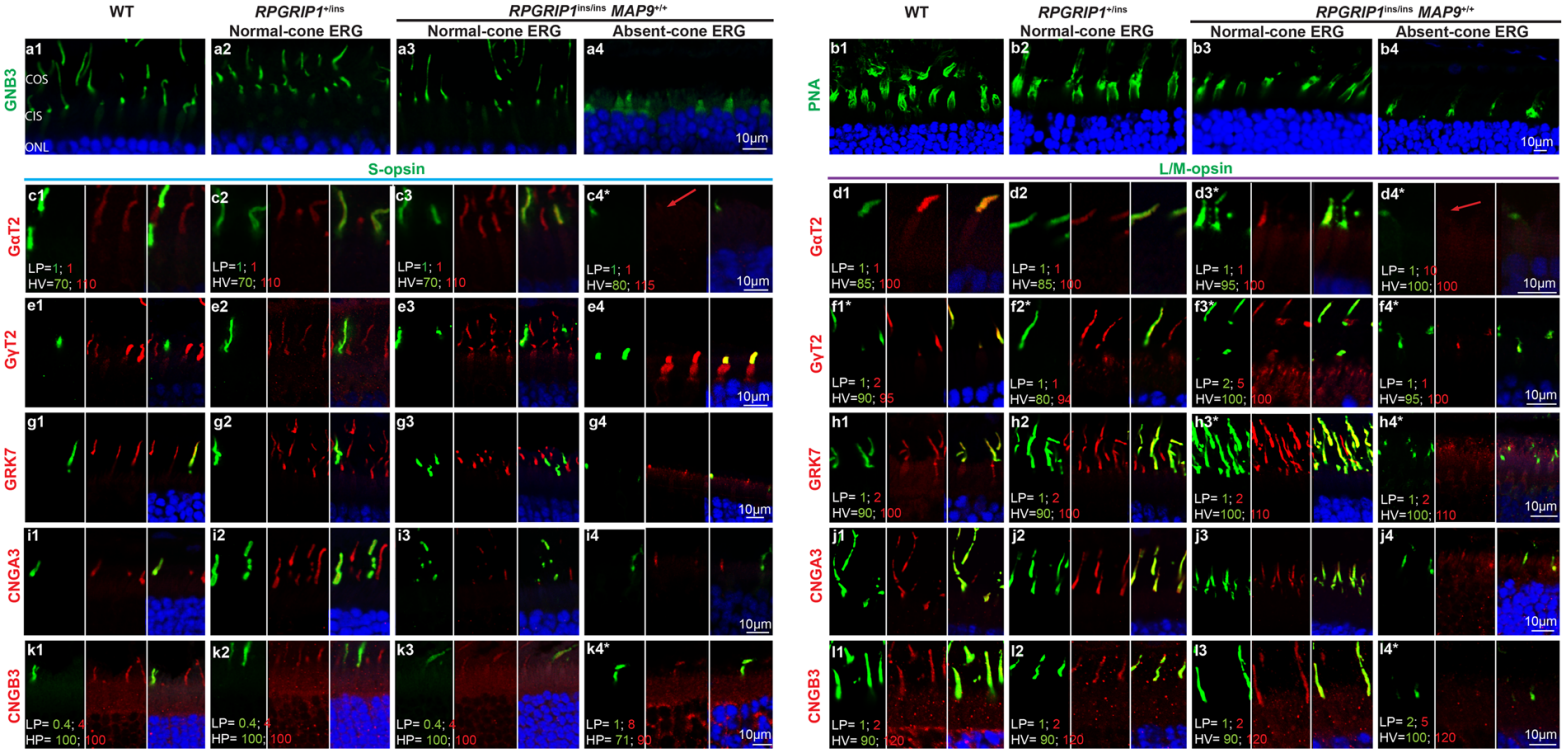
Representative immunolabelling show reduction/absence of some cone transduction proteins. (**a**) Under confocal microscopy, the representative cone ERG^absent^ *RPGRIP1* ^*i*ns/ins^ retina (a4) had faint GNB3 labelling of both of the shortened OS and IS while the other samples have distinct cone OS-specific labelling (a1–3). (**b**) PNA labelling showed cone matrix sheath in all samples groups. In the cone ERG^absent^ retina, the sheaths appeared irregular corresponding to the abnormal cone structure (b4). (**c–l**) Double-immunolabelling with cone OS proteins and S- or L/M-opsin. Each image comprises of green and red channels, and the merged image with DAPI. For WT and normal *RPGRIP1* ^*i*ns/ins^ retinas, all the proteins co-localized with S- or L/M- opsin at the cone OS (1–3 of c–l). However, immunolabelling of these proteins in the cone ERG^absent^ *RPGRIP1* ^*i*ns/ins^ retina was reduced at the OS and occasionally also labelled the IS (e4). In particular, GαT2 labelling was markedly reduced in both S- and L/M-cones (c4, d4: red arrows). CNGA3 was faintly labelled in the OS of L/M- and S-cones (i4, j4) while CNGB3 labelling was hardly detectable in S-cones (k4) and absent in L/M-cones (l4). *In some cases (**c**,**d**,**f**,**h**,**l**), images of the 4 samples labelled with the same antibodies could not be taken under the same illuminating condition. In those instances the imaging conditions for both the green and red channels are noted in the images. COS, cone outer segment; CIS, cone inner segment; ONL, outer nuclear layer, LP, laser power; HV, High voltage.

We next assessed the expression and subcellular localization of known cone OS proteins, starting with those involved in cone phototransduction. Immunolabelling of GNB3 in the cone ERG^absent^ *RPGRIP1* ^ins/ins^ retina showed not only OS but also IS labelling (Fig. 5a4) while the other retinal samples showed OS labelling only (Figs 5a1–3). Immunolabelling of GαT2 in the cone ERG^absent^ *RPGRIP1* ^ins/ins^ retina was severely reduced in both S- and L/M-cones (Fig. 5c,d). GγT2 showed distinct mislocalisation to the IS (Fig. 5e4,f4). GRK7 labelling of cone OS appeared faint in the cone ERG^absent^ *RPGRIP1* ^ins/ins^ retina, with mislocalisation to the IS (Fig. 5g4,h4). Among the cone cGMP-gated channel proteins studied, CNGA3 was faintly labelled in the OS of both cone types of the cone ERG^absent^ *RPGRIP1* ^ins/ins^ retina (Fig. 5i4,j4) while CNGB3 labelling was positive in S- but not in L/M-cones (Fig. 5k4,l4).

**Figure 6:**
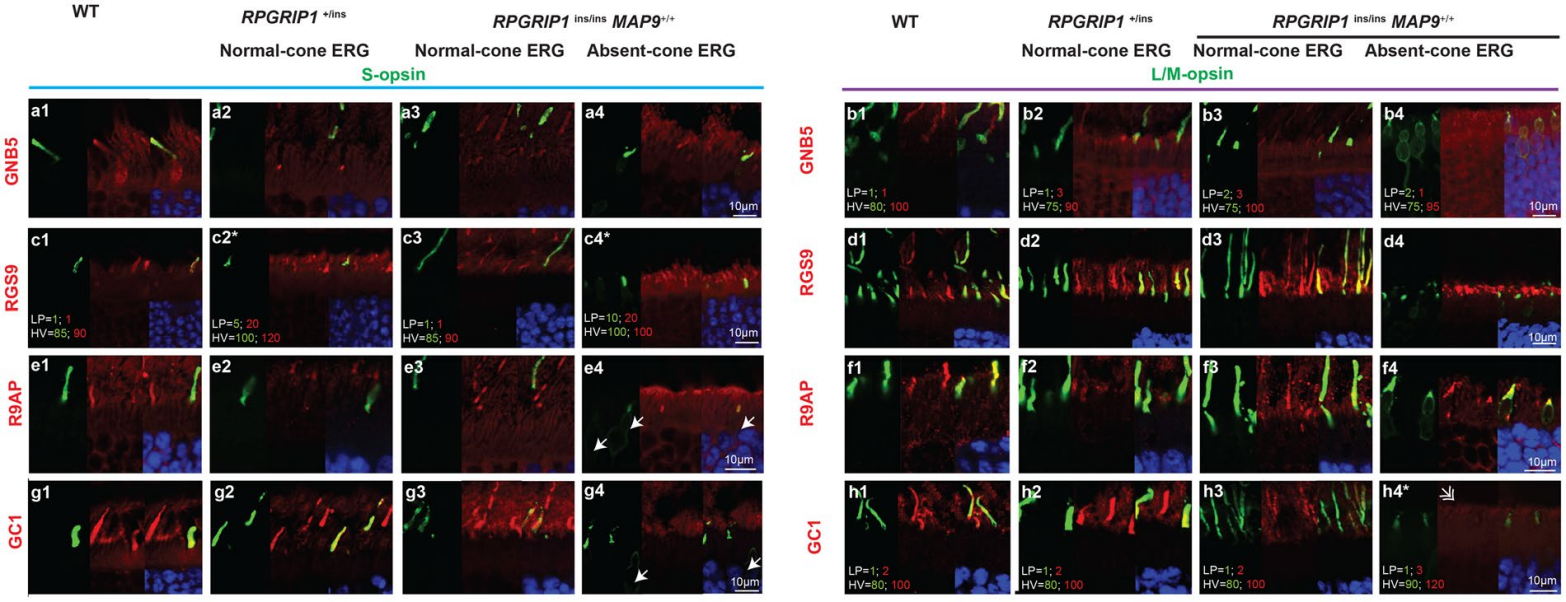
Cone ERG^absent^ retina lacks immunolabelling of GC1 but not of other photoreceptor proteins. Retinal cryosections were labelled with photoreceptor proteins, co-labelled with S-opsin or L/M-opsin for the same set of animals used in Fig. 5, and imaged in confocal microscopy. Each image comprises of green, and red channels, and the merged image with DAPI. In most cases, equal condition was applied to image the pictures for all 4 retinal samples per double-immunolabelling. Occasionally, different conditions for laser power (LP) and high voltage (HV) were used so that protein localization could be visualized in samples with low labelling intensities (*). In such cases, the settings used for imaging the green and red channels are indicated in the picture (**b**,**c**,**h**). (**a**–**f**) Representative images showing labelling of GNB5, RGS9, and R9AP in the OS of rods and cones (S and L/M) in all four retinas. (**g**,**h**) GC1 localized in the OS of rods and both cone types in WT and normal *RPGRIP1* ^*i*ns/ins^ retinas. However, the cone ERG^absent^ *RPGRIP1* ^*i*ns/ins^ retina lacked distinct GC1 co-labelling with either cone types (g4, h4). GC1 labelling of rod OS in h4 is indicated by a white double arrowhead. Rare S-opsin mislocalization to the cell body are indicated by white arrows (e4, g4).

Next, proteins abundantly expressed in photoreceptor OS that are involved in both rod and cone phototransduction were studied (Fig. 6). These included GNB5 (G-protein b subunit 5) that stabilizes the GAP (GTPase-activating protein) complex, RGS9 (GTPase-activating protein regulator of G-protein signalling 9) and R9AP (RGS9-anchoring protein). All three proteins labelled the OS of both rods and cones (S- and L/M-) for all the retinal samples (Fig. 6a–f). Labelling of GC1 was also present in both rods and cones for WT and cone-ERG normal retinas (Fig. 6g1–3,h1–3). However, GC1 labelling in the cone ERG^absent^ *RPGRIP1* ^ins/ins^ retina was extinguished in the cone cells while the rod OS labelling was mildly reduced (Fig. 6g4,h4).

To study the structure of the cone matrix sheath, retinal sections of the cone ERG^absent^ retina and controls were labelled with peanut agglutinin (PNA). Although the cone ERG^absent^ retina showed positive labelling, the labelling appeared fuzzy and the cone sheath was diminished, consistent with the presence of cones with shortened cone OS (Fig. 5b).

### Positive photoreceptor sensory cilia labelling of cilia proteins in all the retinal samples

**Figure 7:**
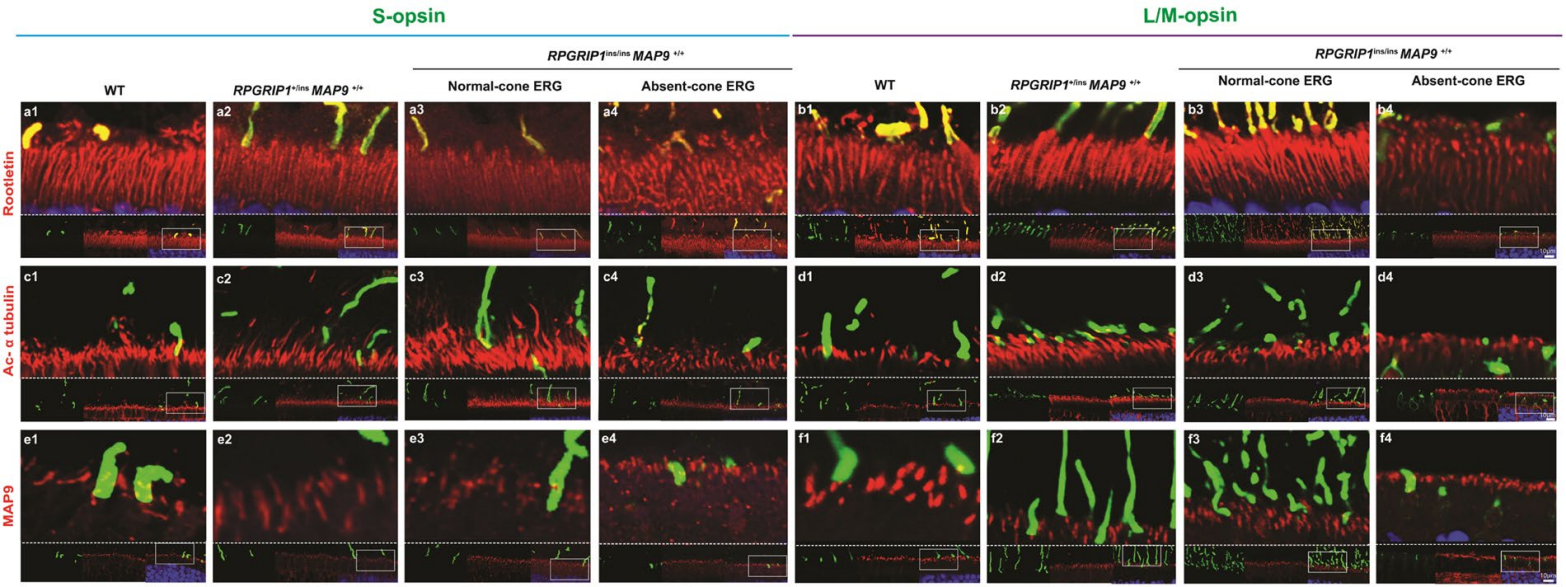
Shorter photoreceptor sensory cilium in the cone ERG^absent^ *RPGRIP1* ^ins/ins^ retina. Retinal cryosections from the same set of animals used in Fig. 5 were labelled with ciliary proteins rootletin (**a**,**b**), acetylated α-tubulin (**c**,**d**), and MAP9 (**e**,**f**), co-labelled with S-opsin or L/M-opsin, and imaged in confocal microscopy. The lower part of each representative image comprises of green and red channels, and the merged image with DAPI. The upper part of the panel shows a magnified version of the area outlined in the lower merged image.

Proper functioning of photoreceptors requires continual transport of proteins, lipids, and metabolites from the IS to OS through the cilia, and its disruption can impact cone-derived photopic vision. RPGRIP1 has been localized to the cilia and is thought to function by anchoring key trafficking proteins to the cilia. Therefore, ciliary structures were studied using a panel of ciliary markers. Rootletin, which is a structural component of the ciliary rootlet and supports the basal bodies and the cilia, was found in the transition zone and photoreceptor OS in all the retinal samples (Fig. 7a,b). Comparable labelling patterns across the different retinal samples were observed for additional ciliary proteins including acetylated α tubulin (Fig. 7c,d) and MAP9 (Fig. 7e,f). Immunolabelling of acetylated α tubulin appeared to correspond to the transition zone as well as the OS axoneme while MAP9 labelled the transition zone only. The length of labelling by some ciliary markers, namely acetylated α tubulin and MAP9 appeared generally shorter in the cone ERG^absent^ retina compared to *RPGRIP1* ^+/ins^ or normal *RPGRIP1* ^ins/ins^ for both L/M- and S-cones (Fig. 7c4–f4). However, artifactual retinal separation from the RPE leading to OS/IS distortion limited the evaluation of the ciliary length. While the implication of potentially shorter cilia in the disease is unclear, its association with cone dysfunction implies that it may disrupt normal ciliary trafficking.

### Normal non-ciliary vesicular transport and dendritic terminals with no inner retinal stress in the cone ERG^absent^*RPGRIP1*^ins/ins^ retina

To determine if mislocalization of the cone opsins from the OS was due to a general defect in vesicular trafficking, another class of vesicles associated with the synapse was studied. Antibodies against proteins localized at the synaptic terminals (synaptophysin, SNAP25 and CtBP2) showed typical punctate labelling of synaptic boutons in both the outer (OPL) and inner (IPL) plexiform layers of all the retinal samples (Supplemental Fig. S2a–c), indicating normal formation of the synaptic vesicles. As glial cells play a critical role in maintaining retinal structure and function, and are one of the first responders to retinal stress, we next labelled retinal sections from all the groups with an antibody against the stress-induced protein, glial fibrillary acidic protein (GFAP). GFAP expression pattern was comparable in all the retinal samples, indicating absence of inner retinal stress at the early age studied (Supplemental Fig. S2b). Further, dual-labelling with antibodies against Goα and PKCα that are markers of ON- and rod-bipolar cells, respectively, showed comparable number of rod and cone-BCs in all the retinal samples. There was no apparent retraction of dendritic terminals (Supplemental Fig. S2d), and the structure of secondary neurons appeared normal.

### Changes in protein and mRNA levels among selected retinal genes

**Figure 8:**
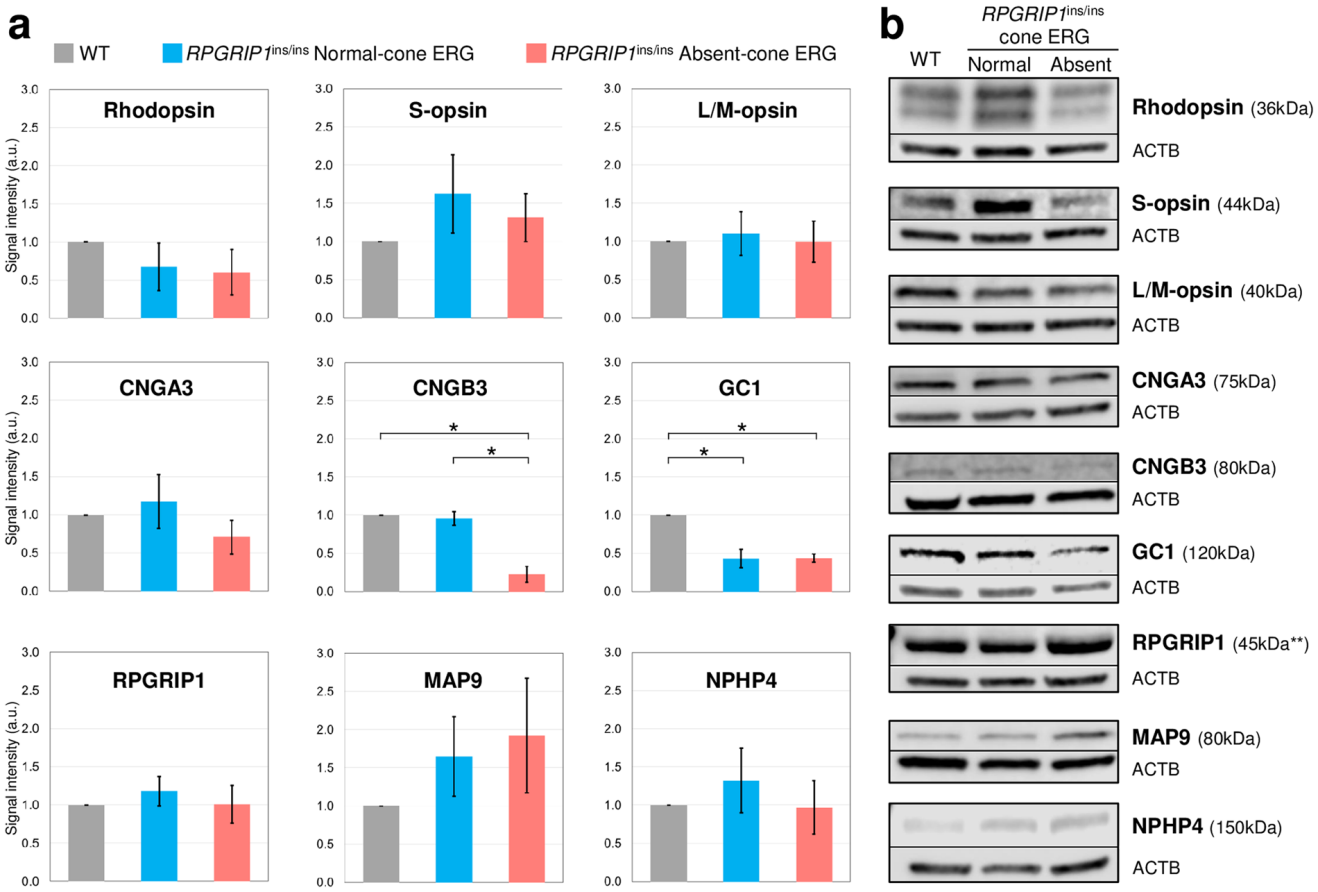
Comparable expression of photoreceptor proteins except CNGB3 and GC1 by Western blot. Western blot of selected photoreceptor proteins were performed using retinal protein extracts from WT (n = 3) and *RPGRIP1* ^ins/ins^ mutants that were either normal (n = 3) or cone ERG^absent^ (n = 3). (**a**) Quantitation of proteins on western blot performed with Li-COR Odyssey software showed significant down-regulation of CNGB3 in the cone ERG^absent^ *RPGRIP1* ^ins/ins^ retina compared to WT (*p = 0.004) or normal *RPGRIP1* ^ins/ins^ retina (*p = 0.011), and of GC1 in the normal (*p = 0.009) and cone ERG^absent^ (*p = 0.0005) *RPGRIP1* ^ins/ins^ retina with respect to the WT. Error bars represent standard errors. (**b**) Representative western blot images for each WT, *RPGRIP1* ^ins/ins^ with normal or cone ERG^absent^ retinas for each of the marker studied are shown side by side along with internal control ACTB. The molecular weight (kDa) of specific immunolabelled bands is indicated.**Anti-canine RPGRIP1 antibody designed to recognise the C-terminus immunolabelled a smaller isoform compared to the expected full-length protein of ~160 kDa. The images of bands for the target protein and ACTB are taken from the same blot and each image has been cropped as delineated by black dividing lines, as well as adjusted for image intensity for optimal visualization.

To quantitate the expression levels in selected retinal proteins that represent the different retinal cells or compartments (i.e. cones and/or rods, cilia), Western blot was performed using WT (n = 3) and *RPGRIP1* ^ins/ins^ (3 normal, 3 cone ERG^absent^) retinas (Fig. 8). In agreement with the IHC findings, the expression of photopigments (Rhodopsin, S- and L/M-opsins) were comparable between the different sample groups. Of note, expression of rhodopsin (*RHO*) was reduced at the mRNA level in 2 of the 3 cone ERG^absent^ samples, compared to WT or normal *RPGRIP1* ^ins/ins^ retinas (Supplemental Fig. S3). However, quantitation at the protein level revealed minimal reduction (Fig. 8). Of the cone-specific proteins, CNGA3 expression was comparable across the groups while CNGB3 was significantly down-regulated in the cone ERG^absent^ retina, consistent with the lack of CNGB3 immunolabelling in the more numerous L/M-cones (Fig. 5l4: faint CNGB3 immunolabelling visible with increased laser intensity shows colocallizing with L/M-opsin). GC1, expressed in both rods and cones, was significantly down-regulated not only in the cone ERG^absent^ *RPGRIP1* ^ins/ins^ retinas but also in the normal *RPGRIP1* ^ins/ins^. The reason for the inconsistency between the IHC and Western data for the latter group is unclear. Finally, ciliary proteins RPGRIP1 and MAP9 whose mutation have been associated with cord1, and NPHP4 which is an interacting protein of RPGRIP1 were quantitated, revealing no differences in expression between the different sample groups. Where data is available, quantitative analysis of mRNA and proteins are mostly in line with the IHC findings including significant reduction in CNGB3 and GC1 in the cone ERG^absent^ mutant. Next, mRNA levels of genes expressed in specific types of retinal cells were quantitated for a panel of genes including rod-only and cone-only expressed genes, as well as those expressed in synaptic terminals (*SNAP25*), and in photoreceptors and bipolar cells (*GNB5, RGS7BP*, *RGS9BP* and *RGS11*). WT (n = 3) and *RPGRIP1* ^ins/ins^ *MAP9* ^+/+^ (2 normal, 3 cone ERG^absent^) retinas were used to examine any alteration associated with the phenotype. While qRT-PCR revealed substantial intra-group variation, there was no significant difference in expression levels across the different sample groups for any of the selected genes (Supplemental Fig. S3). Still, in concordance with the protein expression levels, *CNGB3* had overall decreased mRNA expression in the cone ERG^absent^ *RPGRIP1* ^ins/ins^ mutants. Likewise, *GUCY2D* that encondes GC1 had overall decreased mRNA in the both normal and cone ERG^absent^ *RPGRIP1* ^ins/ins^ mutants.

## Discussion

The *RPGRIP1* ^ins/ins^ mutation was previously associated with a canine CRD^6^ in a closed research colony, characterized by diminished cone ERG^5^ within months after birth, followed by loss of rod photoreceptor functions, leading to complete blindness by 2 years of age^4^. More recently, among the *RPGRIP1* ^ins/ins^ animals in the general breed population, a *MAP9* ^del/del^ mutation has been identified as a modifier leading to early onset of the disease^9,10^. Although the revised digenic (*RPGRIP1* and *MAP9*) model initially seemed to explain the broad range of disease onset in the general breed population, our study herein of an extended *RPGRIP1* ^ins/ins^ canine colony found striking variability in cone-ERG ranging from normal, reduced to absent while retaining normal rod function. These variable cone ERG features were present at the earliest age ERG could be performed in puppies, and remained largely unchanged when repeated over time, with only minor fluctuations in cone ERG amplitudes. The variation in cone ERG phenotype in *RPGRIP1* ^ins/ins^ animals did not appear to be associated with their *MAP9* genotype (discussed later), suggesting an additional genetic mechanism contributing to the observed phenotypic variation.

We expected that photopic vison of cone ERG^absent^ mutants would be defective based on the lack of cone photoreceptor response using full-field photopic ERG. Interestingly, however, vision-guided behaviour test performed in a cone ERG^absent^ animal and its normal and genotype-matched sibling demonstrated comparable results, even at a high light intensity where cones are predominantly used for vision. Of note, *CNGB3*-achromatopsia dogs with complete cone dysfunction have significant defects in navigation at high light intensity when assessed in the same obstacle course^11^. The comparable behaviour between the cone ERG^absent^ and normal *RPGRIP1* ^ins/ins^ littermate remained stable for up to 5.5 years based on repeated obstacle course trials (ref.^8^ and present study). Our findings indicate that the cone ERG^absent^ mutants have residual cone function that is under the threshold of full-field cone ERG, but surprisingly permits photopic vision sufficient for navigation.

Along with the functional ERG assessment of the retina, we also examined morphological changes of the retina that are clinically observable by ophthalmoscopy or sdOCT. Morphological changes typically emerge later than ERG abnormalities, which are commonly observed at the early stage of retinal degenerative diseases. Some of the cone ERG^absent^ *RPGRIP1* ^ins/ins^ eyes did develop ophthalmoscopic abnormalities over time when followed up long term. Of the changes observed, increased reflectivity of the tapetal fundus, multifocal depigmentation in the non-tapetal fundus, and attenuation of retinal vasculature are hallmarks of inherited retinal degenerative diseases in canines. However, the most striking change observed in the cone ERG^absent^ *RPGRIP1* ^ins/ins^ animals was the presence of dark discolouration of the tapetal fundus, which may represent retinal thinning, pigment migration, or a combination of both. While sd-OCT scanning of these dark discoloured regions indicated mild to moderate thinning of the ONL, the disease process at the cellular level awaits future histologic and immunohistochemical examination of retinas with advanced disease.

To study the clinically observed retinal changes at the cellular level, we examined retinal cryosections of WT, *RPGRIP1* ^+/ins^ and *RPGRIP1* ^ins/ins^ (normal and cone ERG^absent^) in young animals, at age 9–11 months. H&E histology revealed comparable thickness of the retina and ONL across the samples studied, consistent with the sd-OCT findings at this age group. However, immunolabelling of specific retinal cells and their protein components revealed striking changes in the cone ERG^absent^ *RPGRIP1* ^ins/ins^ retina. Morphologically, both L/M- and S-cones were markedly shortened in the cone ERG^absent^ *RPGRIP1* ^ins/ins^ retina, with shorter IS, OS and photoreceptor sensory cilia. In addition, while the overall expressions of L/M- and S-opsins were comparable quantitatively across the different retinal samples studied by Western, IHC revealed extensive L/M-opsin mislocalization to the cell membranes of the IS, soma and synaptic pedicles in the cone ERG^absent^ *RPGRIP1* ^ins/ins^ retina, as assessed by confocal microscopy. These indicate: A) defective protein trafficking from the L/M-cone cell body through the IS and cilia to the OS giving rise to a small OS; B) primary formation of shortened cone OS that is not capable of accommodating normal levels of proteins resulting in diversion of L/M-opsin to elsewhere within the L/M-cone, or a combination of both, providing a potential explanation for cone dysfunction. Of note, the S-opsins of the less numerous S-cones were only rarely mislocalized and to a lesser degree, indicating a primary L/M-cone defect in the cone ERG^absent^ *RPGRIP1* ^ins/ins^ animals. As these findings suggested that the intact S-cones might provide sufficient photopic vision for navigation, L/M- and S-cone functions were tested separately using chromatic ERGs. Results showed that S- and L/M-cone derived ERGs were equally affected in the cone ERG^absent^ and cone ERG^reduced^ *RPGRIP1* ^ins/ins^ animal (Supplemental Fig. S1). These results, together with the shorter morphology observed in both cone types, support a pathogenesis that affects both L/M- and S-cones.

Our immunolabelling studies further revealed that the level of specific cone transduction proteins was reduced in the cone ERG^absent^ *RPGRIP1* ^ins/ins^ retina, with CNGB3 being undetectable in L/M-cones and only faintly labelled in S-cones. Western blot analysis showed significantly reduced expression of CNGB3 in the cone ERG^absent^ *RPGRIP1* ^ins/ins^ retinas (p ≤ 0.011), supporting the IHC findings. Mutations in *CNGB3*, the beta subunit of the cyclic nucleotide-gated channels in cones, are associated with loss of cone function leading to achromatopsia (day blindness) in humans and in certain canine breeds^11–14^. However, our cone ERG^absent^ *RPGRIP1* ^ins/ins^ animal retains sufficient photopic vision, suggesting that the observed reduction in CNGB3 is a secondary effect of the diseased cones, rather than a primary defect as in *CNGB3*-achromatopsia. Loss of CNGB3 has been associated with loss of its heterotetrameric partner CNGA3 since it regulates the expression of CNGB3 as was shown in the mouse model of CNGA3-achromatopsia before and after gene therapy^15^. However, the role of CNGA3 primarily down-regulating CNGB3 in the cone ERG^absent^ *RPGRIP1* ^ins/ins^ retina is less likely as our IHC and Western data show that CNGA3 is not significantly affected.

We also studied the expression of proteins that are generally expressed in both rods and cones. Of the proteins assessed, labelling of GC1 in the cone ERG^absent^ retina was undetectable in both cone types while the rods had normal GC1 labelling. Western blot analysis showed significantly reduced overall GC1 expression in the cone ERG^absent^ retina reflecting the GC1 loss. GC1 is encoded by *GUCY2D* and restores intracellular cGMP levels and allows reopening of cGMP-gated cation channels in both rods and cones. While *GUCY2D* that encodes for GC1 is one of the most frequently mutated genes associated with Leber congenital amaurosis^16–18^, *GUCY2D* mutations have also been associated with cone-rod dystrophy 6 (CORD6) in humans^19–21^. GC1 stabilizes cone transducin and cone PDE^22^ but the mechanism by which defects in GC1 primarily affect cone function in CORD6 patients remains unexplained. The cone-specific loss of GC1 in the cone ERG^absent^ *RPGRIP1* ^ins/ins^ retina may be a secondary change in response to the cone morphological and functional abnormalities, or may reflect a primary genetic defect in *GUCY2D*. Genome-wide mapping and sequencing to search for genetic changes associated with the cone ERG defect are under way and will help determine if changes in GC1 contribute to the phenotypic variability observed in our *RPGRIP1* ^ins/ins^ colony.

Finally, known cilia markers were assessed to determine if there were potential alterations in morphology that may impact ciliary function. The cilia were of particular interest because the two disease-associated genes *RPGRIP1* and *MAP9* are both localized to the cilia^21,23^. Microtubules (MTs) are important elements of the ciliary structure and cytoskeletons. MT acetylation causes the motor proteins kinesin and dynein to bind to MTs thereby aiding in cellular transport^24^. Examination of the MT kinetics focusing on the balance between acetylation and de-acetylation of cilia in an ERG^absent^ *RPGRIP1* ^ins/ins^ background might shed light on the molecular pathogenesis. While the role of MAP9, one of many microtubule-associated proteins (MAPs), in retinal homeostasis and function remains to be established, our IHC data show localization of MAP9 in the transition zone of the cilia of both rods and cones. Localization of RPGRIP1, thought to anchor the protein complexes^25^, to the same region as MAP9 supports a potential role for MAP9 as a modifier of RPGRIP1 function.

To quantitate the expression of some of the photoreceptor-expressed proteins, Western blotting analysis was carried out. The findings were largely in line with that of IHC that showed significantly reduced expression of CNGB3 and GC1 in the cone ERG^absent^ *RPGRIP1* ^ins/ins^ retina. The cilia proteins, including RPGRIP1 and MAP9 that are associated with cord1, were also examined by Western blot revealing comparable expression between WT and *RPGRIP1* ^ins/ins^ retinas both normal and cone ERG^absent^. It is important to note that our custom anti-canine RPGRIP1 antibody against the C-terminal RID domain, identified a single product of 45 kDa which corresponds to a truncated isoform, rather than the full-length ~160 kDa product. As the expression pattern was comparable regardless of the presence or absence of the *RPGRIP1* ^ins/ins^ mutation, the truncated product likely represents an RPGRIP1 isoform that does not utilize the insertion mutation site but includes the C-terminal RID against which the custom canine antibody was designed to recognize. The presence of smaller isoforms is supported by previous studies where extensive *RPGRIP1* isoforms were identified arising from complex splice variants as well as alternative start codons found in the retina of dogs^26,27^ and in other species including humans^28^. In particular, an isoform with an alternative start codon (chr15:18,352,430–18,352,432; CanFam3.1) in canine exon 13 as verified by 5′ RACE experiments^27^ leads to a truncated product corresponding to that identified in our current study. While the predicted full-length transcript of ~160 kDa encompassing most of the exons was not identified by our custom antibody, Castagnet *et al*. was able to detect a ~160 kDa product using an antibody (Ab38) that was also designed to recognize the domain RID, albeit with longer exposure of the blot^28^. That antibody, when used in canine retinal protein extracts, only identified the lower molecular weight isoforms^8^, and it is possible that the differences in extraction methods of retinal proteins for Western analysis are responsible. Based on these findings, studies are ongoing to investigate the role of the truncated RID-containing isoform, as well as the basis of the technical challenges in capturing the full-length product both at the transcript and protein levels.

While assessment of the protein expression was limited by the availability of antibodies that cross-react with canine tissue, retinal transcript levels of a panel of genes preferentially expressed in either rods, cones, or synaptic terminals were assessed by qRT-PCR. For the genes expressed in rods, cones, and synaptic terminals studied, there was no difference at the mRNA level between WT and normal or cone ERG^absent^ *RPGRIP1* ^ins/ins^ retinas. This indicates that transcript expression of the selected genes was not affected by the functional and morphological changes of cones in the cone ERG^absent^ retina. While only a few selected retinally expressed genes are studied herein, global changes in retinal transcripts are being investigated by total transcriptomic study. This on-going approach will not only extend the current qRT-PCR data to a comprehensive profile of the transcriptome, but will also aid in identifying transcripts that might contribute to the variation in cone ERG phenotype that we observe.

As the current research colony was being established, we primarily focused on generating *RPGRIP1* ^ins/ins^ animals, allowing the *MAP9* mutation to segregate naturally. Of the eight double homozygotes (*RPGRIP1* ^ins/ins^ *MAP9* ^del/del^) that were generated, all exhibited a cone ERG^absent^ phenotype with early and rapidly-progressing retinal changes, suggesting that the *MAP9* ^del/del^ modifier might have a role in exacerbating the retinal phenotype. However, as *RPGRIP1* ^ins/ins^ animals of any *MAP9* genotypes were found to show the cone ERG^absent^ phenotype in the current colony, we conclude that *MAP9* ^del/del^ is not essential for the development of an abnormal cone ERG phenotype. Based on the morphological data in the cone ERG^absent^ retina, we hypothesize that an additional gene product that contributes to the formation or maintenance of cone morphology is associated with the cone ERG phenotype. This locus may have been present but fixed in the original research colony leading to a more severe phenotype (the “classic” cord1), and hence only a single strong linkage in the first study. Later on when we bred dogs from outside this original colony and one female impregnated with the semen of Casper from the original colony to develop our own colony of dogs, events of recombination occurred at the fixed genotype and the third gene naturally segregated with the cone ERG phenotype. Alternatively, outcrossing in our current colony may have introduced a new locus that has a protective or alleviating effect. We will examine these hypotheses once the locus corresponding to the cone ERG phenotype is mapped and candidate gene mutation identified in our on-going studies.

In conclusion, *RPGRIP1* ^ins/ins^ animals in the canine colony reported herein show a spectrum of phenotypes ranging from normal, cone ERG^reduced^, to cone ERG^absent^ that does not segregate with the known modifier *MAP9*. Morphologically, the cone ERG^absent^ *RPGRIP1* ^ins/ins^ retina has shortened cones, accompanied by down-regulation of key transduction proteins such as CNGB3 and GC1. While *RPGRIP1* ^ins/ins^ and *MAP9* ^del/del^ have previously been associated with cord1 and their roles in disease pathogenesis remain important, neither one alone is sufficient to manifest the disease. The involvement of an additional locus responsible for the cone morphological and functional defects is indicated, and its identification will allow a unique opportunity using this canine model to tease out the pathogenesis and gene/protein interactions of a multigenic form of inherited retinopathy that has been a challenge to study in genetically diverse human populations.

## Material and Methods

### Ethics statement

The research was conducted in full compliance and strict accordance with the Association for Research in Vision and Ophthalmology (ARVO) Resolution on the Use of Animals in Ophthalmic and Vision Research, and the Policies on the Use of Animals and Humans in Neuroscience Research of the Society of Neuroscience. The protocol was approved by the host institute’s IACUC (number: 801870). While none of the procedures were expected to cause discomfort, all efforts were made to minimize potential suffering of the animals.

### Colony establishment

The canine research colony was established by outcrossing two unrelated purebred *RPGRIP1* ^ins/ins^ MLHDs to unrelated mix breed dogs, and subsequently expanded by backcrossing and inter-crossing (Fig. 1). The colony was maintained at the Retinal Disease Studies Facility at the University of Pennsylvania. Briefly, semen from an *RPGRIP1* ^ins/ins^ dog (Casper), obtained from the previously described cord1 research colony at the Animal Health Trust, Lanwades Park, Newmarket, UK^4,6^ was used to inseminate one normal female (N220). The mutant founder had typical cord1 features and showed early-onset generalized retinal degeneration with diminished 30-Hz cone flicker ERG response that further decayed in the first year of life^5^. An unrelated female *RPGRIP1* ^ins/ins^ MLHD (R10) affected with advanced end-stage retinal degeneration at 2.6 yrs was the second founder of the colony. Breeding with a male *RPGRIP1* ^+/+^ MLHD (R9) resulted in a litter (R11-R15) of heterozygotes having clinically normal vision and ERG. Subsequent breeding among the F1 progeny gave rise to *RPGRIP1* ^ins/ins^ mutants^8^.

### Animals

Sixty-four related dogs from the purpose-bred cord1 research colony (Fig. 1) were included in the study. In addition, WT normal dogs unrelated to the colony were included for IHC (n = 3) and qRT-PCR (n = 3). All the animals were genotyped for the presence of *RPGRIP1* ^ins/ins^ and *MAP9* mutations (Fig. 1) as previously described^6,10^. The pedigree illustrates the genotypes at *RPGRIP1* and *MAP9* loci and the ERG functions studied.

### Ophthalmic examinations

Each dog underwent comprehensive ophthalmic examinations at 6–18 wks, and approximately every 6 months thereafter. The fundus was examined by indirect ophthalmoscopy. A hand-held fundus camera (RC-2; Kowa Ltd, Nagoya, Japan) or a contact retinal camera (RetCam Shuttle; Clarity Medical Systems, Pleasanton, CA, USA) was used for fundus photography.

### Electroretinography (ERG)

Following pupillary dilation, full-field flash ERG was performed as detailed previously^29^, on both eyes under general inhalation anaesthesia (induction with IV propofol; maintenance with isofluorane) using a custom-built Ganzfeld dome fitted with the LED stimuli of a ColorDome stimulator (Diagnosys LLC, Lowell, MA, USA). To independently assess L/M- and S-cone functions, light adapted full-field ERGs were performed using different colour background lights and light flashes. The background was produced by LED lights at an intensity of 32.35 (S) cd*s/m^2^, and the flashes were produced by LED lights of increasing intensity. Briefly, L/M-cone mediated responses were elicited by using increasing intensities of yellow light flashes (3.235, 16.18, and 22.65 (S) cd*s/m^2^) on a blue background, followed by increasing intensities of green light flashes (3.235, 16.18, and 22.65 (S) cd*s/m^2^) also on a blue background. S-cone mediated responses were elicited by using increasing intensities of blue flashes (3.235, 16.18, and 22.65 (S) cd*s/m^2^) on white followed by yellow backgrounds. Animals were adapted for each new background colour for 10 min.

### Visual Behaviour Testing

A 3.6 m-long custom made obstacle course was used to evaluate visually guided behaviour as described previously^30^, under ambient (0.2, 1, 10, 65, and 646 lux) and lower (0.003, 0.009, and 0.2 lux) illuminations. The obstacle panels were changed each time a new test run was performed. Two *RPGRIP1* ^ins/ins^ littermates, one with normal and the other cone ERG^absent^, were initially tested at 4 mon. The performance was previously reported from 6 through 10 mon^8^. Here the long-term follow up (over a 5.5-year period) of the animals have been studied with assessments done every 1–6 months.

### Spectral domain optical coherence tomography (sd-OCT)

*In vivo* imaging was done using the same general anaesthesia protocol by using a combined cSLO/sd-OCT instrument (Spectralis HRA + OCT, Heidelberg, Germany). Briefly, overlapping *en face* images of reflectivity with near-infrared illumination (820 nm) were obtained with a 55° lens to delineate fundus features such as optic nerve, retinal blood vessels, and other local changes. A 30° lens was used to perform sd-OCT with linear and raster scans. Overlapping 30° × 20° raster scans (120 µm (9 ART) × 49 sections) were recorded covering large regions of the retina. The outer nuclear layer thickness was measured by manual segmentation using the analysis tool that accompanied the instrument software.

### Tissue collection

Retinal tissues were promptly harvested from selected dogs following euthanasia (Euthasol; Virbac, Fort Worth, TX, USA). Posterior eye cups were collected for histology and IHC from a group of age-matched dogs (9–11 mon): 1 *RPGRIP1* ^+/ins^, 2 normal and 3 cone ERG^absent^ *RPGRIP1* ^ins/ins^, and 3 WT control dogs (2–5 yrs) unrelated to the research colony.

### Histology and IHC

The posterior eye cups were processed as previously described^31^. H&E-stained 10 µm sections from the superior quadrant were used to quantitate ONL and INL thickness (µm) and cell counts were indicated by rows of nuclei. Beginning 1,000 µm from the ora serrata, and extending centrally at 1,000 µm intervals, the number of rows of nuclei was determined in three areas of a 558μm length visual field.

For IHC, cryosections were incubated overnight with appropriate primary antibodies (Supplemental Table 1) in dilution buffer (4.5% fish gelatin, 0.1% sodium azide and 5% BSA in PBST). Heat-induced epitope retrieval was performed in all cases (except PNA) prior to primary antibody treatment by incubating the sections in citrate buffer (pH = 6) at 125 °C for 10 min in a pressure cooker. In all cases, antigen-antibody complexes were visualized with fluorochrome-labelled secondary antibodies (Alexa Fluor Dyes, Molecular Probes, Invitrogen, Carlsbad, CA, USA) with 1:200 dilution. Slides were examined under 20X and 40X objectives on an epifluorescence microscope (Axioplan; Carl Zeiss Meditec, Oberkochen, Germany). Images were digitally photographed from the same retinal regions in the 4 samples with a Spot 4.0 camera (Diagnostic Instruments, Inc., MI, USA). Some slides were visualized under Nikon A1R confocal microscope (Nikon Instruments Inc. NY, USA) at 60x and 100x, and digital images were captured and processed using the NIS Elements software (Nikon Instruments Inc.). The settings for capturing images in the microscopes were constant in all samples for each antibody except for those cases where changes were necessary to better visualize the localization of down regulated proteins, and the settings have been indicated in the images.

### TUNEL Assay

To look for cells dying through apoptosis in the cone ERG^absent^ retina, TUNEL assay was done on all quadrants of retinal tissue sections in WT, *RPGRIP1* ^+/ins^ and *RPGRIP1* ^ins/ins^ (normal and cone ERG^absent^) according to the manufacturer’s protocol (*in situ* cell death detection kit; Roche Diagnostics Corporation, IN, USA), stained with DAPI, and visualized under fluorescence microscope.

### Genotyping *RPGRIP1* insertion and *MAP9* deletion

All animals born in the colony were genotyped for *RPGRIP1* insertion (primer sequences listed in Supplemental Table 2a) and *MAP9* deletion using the primer pairs described by Forman *et al*.^10^.

### RNA extraction, cDNA synthesis and quantitative Real time PCR (qRT-PCR)

Whole retinas were isolated from the eyecups within 1–2 min of euthanasia and flash frozen in liquid nitrogen and stored at −80 °C. Retinal RNA was extracted using Trizol method from 3 WT controls (1 M, 2 F; 7–14 mon), normal (2 M, 1 F; 9–11 mon) or cone ERG^absent^ (1 M, 5 F; 9 mon) *RPGRIP1* ^ins/ins^ mutants, DNase I (Ambion, Carlsbad, CA, USA) treated, and reverse-transcribed. Due to limited tissue availability, three of the six available cone ERG^absent^ retinas as indicated as blue asterisk in Fig. 1 was used for a given gene analysis. Briefly, 20ng of cDNA was used in a total reaction volume of 10 μl in a 384-microwell plate and run on a 7900HT fast Real-Time PCR System (Applied Biosystems, Foster City, CA, USA). For each sample, 3 technical replicates were run. Supplemental Table 2b provides details of all the genes and qRT-PCR primers (SYBR-green, TaqMan). Fold change was calculated by ∆∆CT method^32^. Cut off for fold change was set at ≥±2 for differential expression.

### Western blot analysis

Retinal proteins were extracted by standard method using T-PER™ reagent (Thermo Fisher Scientific, Rockford, IL, USA) and quantitated by BCA Protein Assay Kit (Thermo Fisher Scientific). Each sample was denatured in 1x Laemmli sample buffer (Bio-Rad, Hercules, CA)-5% 2-mercaptoethanol at 95 °C for 5 min. 30 µg total protein was loaded in triplicate on a precast gel having 1x SDS/Tris/Glycine buffer. The gel was subsequently wet transferred into a nitrocellulose membrane which was then cut to separate each triplicate samples, blocked with 1x Odyssey PBS Blocking Buffer (LI-COR, Lincoln, NE, USA) for 1 h at room temperature and probed with the primary antibody (Supplemental Table 1a) for 18 h at 4 °C. Membranes were washed with PBST thrice and incubated for 1 h at room temperature in fluorescent-labelled secondary antibodies (LI-COR, Lincoln, NE. USA). Immunoblots were washed in PBST thrice and a final wash in PBS once. Each immunoblot was scanned on LI-COR Odyssey Fc Dual-Mode Imaging System and normalized against β-actin using the LI-COR Image Studio Software. For each sample, the average of the three β-actin-normalized values per each antibody was compared with that of the corresponding normalized and averaged control value and expressed as fold-changes. Statistical significance was calculated by an unpaired t-test using Graphpad.

## Electronic supplementary material

Supplementary data

Suppl video s1a

Suppl video s1b

Suppl video s1c
