# Author Correction: Variabilities in retinal function and structure in a canine model of cone-rod dystrophy associated with *RPGRIP1* support multigenic etiology

**DOI:** 10.1038/s41598-018-31337-1

**Published:** 2018-08-24

**Authors:** Rueben G. Das, Felipe Pompeo Marinho, Simone Iwabe, Evelyn Santana, Kendra Sierra McDaid, Gustavo D. Aguirre, Keiko Miyadera

**Affiliations:** School of Veterinary Medicine, University of Pennsylvania, Philadelphia, PA 19104 USA

Correction to: *Scientific Reports* 10.1038/s41598-017-13112-w, published online 09 October 2017

The Acknowledgements section in this Article is incomplete.

“The authors thank Anuradha Dhingra for assistance with confocal microscopy, José Manuel Guzmán and Valerie Dufour for performing part of ERG and sd-OCT, Kendall Carlin for technical assistance, and Leslie King for reviewing the manuscript. The authors are grateful to William Beltran for providing the hCAR antibody and control retinas, and the following colleagues for antibodies as detailed in the supplementary information; Noga Vardi, András Komáromy, Vadim Arshavsky, Robert Molday, Theodore G. Wensel, Cheryl Craft, Rui Chen, Hemant Khanna, Tiansen Li, and David Sargan. University Research Foundation (URF) from the University of Pennsylvania, NEI/NIH EY-06855 and -17549, Foundation Fighting Blindness (FFB) for Career development grant and Facility grant, Van Sloun Fund for Canine Genetic Research, and Macula Vision Research Foundation.”

should read:

“The authors thank Anuradha Dhingra for assistance with confocal microscopy, José Manuel Guzmán and Valerie Dufour for performing part of ERG and sd-OCT, Kendall Carlin for technical assistance, and Leslie King for reviewing the manuscript. The authors are grateful to William Beltran for providing the hCAR antibody and control retinas, and the following colleagues for antibodies as detailed in the supplementary information; Noga Vardi, András Komáromy, Vadim Arshavsky, Robert Molday, Theodore G. Wensel, Cheryl Craft, Rui Chen, Hemant Khanna, Tiansen Li, and David Sargan. University Research Foundation (URF) from the University of Pennsylvania, NEI/NIH EY-06855 and -17549, Foundation Fighting Blindness (FFB) for Career development grant and Facility grant, Van Sloun Fund for Canine Genetic Research, Macula Vision Research Foundation, and Margaret Q. Landenberger Research Foundation. The paper was also funded by NIH grant P30EY-001583.”

