# Supplementary material for: Variabilities in retinal function and structure in a canine model of cone-rod dystrophy associated with RPGRIP1 support multigenic etiology
Source: Sci Rep. 2017 Oct 9;7:12823. doi: 10.1038/s41598-017-13112-w (PMC5634483; doi:10.1038/s41598-017-13112-w)
Supplement: Supplementary file 1 — Supplementary data [file 41598_2017_13112_MOESM1_ESM.pdf]

Title:

Variabilities in retinal function and structure in a canine model of cone-rod dystrophy associated with *RPGRIP1* supports multigenic etiology

Authors:

Rueben G. Das, Felipe Pompeo Marinho, Simone Iwabe, Evelyn Santana, Kendra Sierra McDaid, Gustavo D. Aguirre, Keiko Miyadera*

Affiliation:

School of Veterinary Medicine, University of Pennsylvania, Philadelphia, PA 19104, USA

Correspondence should be addressed to (kmiya@upenn.edu)

Supplemental table 1a. Antibodies used in the study

Marker	Host	Retinal localization of target protein	Dilution (IHC)	Dilution (Western)	Source
CNGA3	Rabbit	Cone outer segment	1:500	1:500	András Komáromy, Michigan State University
CNGB3	Rabbit	Cone outer segment	1:500	1:1000	András Komáromy, Michigan State University
GαT2	Rabbit	Cone outer segment	1:300	-	Vadim Arshavsky, Duke University
GγT2	Rabbit	Cone outer segment	1:300	-	Vadim Arshavsky, Duke University
GRK7	Rabbit	Cone outer segment	1:300		GeneTex GTX53604
GC1	Mouse	Rod and cone photoreceptors	1:20	1:100	Robert Molday, University of British Columbia
GNB3	Rabbit	Cone photoreceptors and dendrites of islet-1 positive cone ON-bipolar cells	1:100	-	Santa Cruz SC-381
GNB5 (G4718)	Goat	Photoreceptors and dendrites of ON-bipolar cells	1:1,000	-	Theodore Wensel, Baylor College of Medicine
R9AP (G3860)	Goat	Photoreceptors and dendrites of ON-bipolar cells	1:1,000	-	Vadim Arshavsky, Duke University
RGS9-1 (R4433, AA 226-484)	Rabbit	Rod and Cone photoreceptors and dendrites of ON-bipolar cells	1:1,000	-	Theodore Wensel, Baylor College of Medicine
Rootletin	Human	Rootlet of photoreceptor sensory cilium and photoreceptor outer segment	1:400	-	AbD Serotec HCA009
NPHP4	Rabbit	Photoreceptor ciliary transition zone	-	1:500	Santa Cruz SC20204
RPGRIP1 (Miya2)	Rabbit	Photoreceptor connecting cilium	-	1:1,000	David Sargan, University of Cambridge
MAP9	Rabbit	Transition zone of photoreceptor sensory cilium	1:500	-	Novus Biologicals NBP1-90842
	Goat		-	1:500	Santa Cruz SC-164961
Acetylated alpha tubulin	Mouse	Axoneme and transition zone of photoreceptor sensory cilium; microtubules	1:1,000	-	Sigma T7451
L/M-opsin	Rabbit	OS of Red Green cone photoreceptors	1:500	1:2,000	Millipore AB5405
	Goat		1:100		Santa Cruz SC-22117
S-opsin	Rabbit	OS of Blue cone photoreceptors	1:500	1:2,000	Millipore AB5407
	Goat		1:100	-	Santa Cruz SC-14363
Rod opsin	Mouse	Rod photoreceptors	1:300	-	Millipore MAB 5316
	Mouse		-	1:1,000	Millipore MAB 5356
GFAP	Rabbit	Müller cells and astrocytes	1:1,000	-	Dako Z0334
Goα	Mouse	Dendritic tips of ON bipolar cells	1:5,000	-	Millipore MAB 3073
PKCα	Mouse	Rod bipolar cells	1:100	-	BD Transduction Laboratories 610107
CtBP2	Mouse	Photoreceptor and bipolar cell ribbon synapses	1:200	-	BD Bioscience 612044
Synaptophysin	Rabbit	Inner and Outer plexiform layers	1:10,000	-	Dako A0010
SNAP25	Mouse	Photoreceptor synapses and Horizontal cells	1:5,000	-	Millipore MAB331
*PNA lectin (red/green)	NA	Cone matrix sheath	1:20	-	Molecular Probes L-32458 (red)/ L21409 (green)
hCAR	Rabbit	Cone photoreceptors	1:10,000	-	Cheryl Craft, University of South California
	Goat		1:100	-	William Beltran, University of Pennsylvania
ACTB	Mouse	Ubiquitous	-	1:10,000	Millipore MAB1501
	Rabbit			1:10,000	Abcam ab8227

*Antigen retrieval step was not used for IHC.

Supplemental table 1b. Antibodies that did not work in IHC

Marker	Host	Retinal localization of target protein	Source
Centrin3	Mouse	Basal body of photoreceptor connecting cilium	Millipore 04-1624
CEP290	Rabbit	Photoreceptor ciliary transition zone	Proteintech 22490-1-AP
SPATA7	Rabbit	Photoreceptor connecting cilium	Rui Chen, Baylor College of Medicine
SDCCAG8	Rabbit	Photoreceptor connecting cilium	Novus Biologicals NBP2-13288, Proteintech 13471-1-AP
†RPGR	Rabbit	Photoreceptor ciliary transition zone	Hemant Khanna, University of Massachusetts Medical School
NPHP1	Rabbit	Photoreceptor ciliary transition zone	Santa Cruz SC20204
NPHP4	Rabbit	Photoreceptor ciliary transition zone	Santa Cruz SC99012, Proteintech13812-1-AP
KIF3a	Rabbit	basal body of connecting photoreceptor cilium axoneme and synaptic ribbons of cones and rods; IS, Muller and Ganglion cells	Abcam Ab11259, Proteintech13930-1-AP
IFT57	Rabbit	tip of photoreceptor connecting cilia in OS base	NBP1-32932, Novus Biologicals, Proteintech 11083-1-AP
IFT88	Rabbit	tip of photoreceptor connecting cilia in OS base, axoneme and distal part of OS	NBP1-79538, Novus Biologicals
mouse RPGRIP1	Mouse	Photoreceptor connecting cilium	Tiansen Li, National Eye Institute
"human" for mouse RPGRIP1 staining on IHC only	Mouse	Photoreceptor connecting cilium	Tiansen Li, National Eye Institute
RPGRIP1 (Miya1)	Rabbit	Photoreceptor connecting cilium	David Sargan, University of Cambridge
RPGRIP1 (Miya2)	Rabbit	Photoreceptor connecting cilium	David Sargan, University of Cambridge
Human RPGRIP1	Mouse	Photoreceptor connecting cilium	Tiansen Li, National Eye Institute
PDE6α’	Rabbit	Cone OS	Tiensen Li, National Eye Institute

†Anti-RPGR also failed in Western.

Supplemental table 2a. List of PCR primers used in the study

Primer	Gene	Forward primer	Reverse primer	Amplicon (bp)
RPGRIP1_44bpins	*RPGRIP1*	AAGAATATGAAAGCTCAACCACCC	AAAGACTTAAGGAGAACACAAGGTACC	157 (201 insertion)

Primer pairs used by Forman et al. 2016 were used to detect *MAP9* deletion.

Supplemental table 2b. List of qPCR primers used in the study

I. Custom made

Gene	Forward primer	Reverse primer	MGB probe	Amplicon (bp)
OPN1LW	CAGCGTCATCATACTGTGCTACCT	GGACTCAGATTCTTTCTGCTGCTT	6FAM-ATCCGAGCGGTGGC	79
OPN1SW	CGCCATGTTTGTGCTTTGG	GATGACAATGTAGCGCTCAAAGG	6FAM-CTCTACAGCAGGTCTGGTGA	99
RHO	ATGATTGTCATATTCTTCTGCTATGGA	TGGGTGGTGGCCGATTC	6-FAM-CAGTCAAGGAGGCAGCT	187
CNGA3	GCCCTGCCTGTCTTCTATAACTG	CACAGCATCACGTGCTCAGA	6FAM-TGTGCAGGGCCTGTT	83
CNGB3	AAGATCCTGATCCAAGCAATCAG	CTTCAAACGTGACTGGAGTCATCT	6FAM-CTCAGCAATCTACAAGACA	105

II. Taqman Probes

Gene	ID	Catalog #	Amplicon (bp)
GAPDH	Hs02786624_g1	4331182	157
CNGB1	Cf02662052_m1	4351372	124
CNGA1	Cf02628568_m1	4351372	98
SAG	Cf02628845_m1	4351372	78
GNB1	Cf02628964_m1	4448892	82
GNB5	Cf02631024_m1	4351372	92
GRK7	Cf02717515_m1	4351372	153
ARR3	Cf03460116_m1	4331182	67
PDE6C	Cf02652240_m1	4448892	138
GUCY2D	Cf02628896_g1	4448892	95
RGS7BP	Cf00936133_m1	4351372	110
RGS9BP	Cf02678958_s1	4351372	123
RGS11	Cf02660333_m1	4351372	66
SNAP25	Hs00938962_m1	4448892	64

Supplemental table 3. List of animals included in the study

a. Study animals - OCT performed

RPGRIP1	*MAP9*	Dog ID	Sex	ERG Age (m)	ERG Cone amplitude	OCT Age (m)	OCT ONL observation
ins/ins	+/+	R17	M	5	Normal	23	Normal
				22	Normal	49	Normal
				49	Normal	54	Normal
				85	Normal	84	Normal
	+/del	R5	F	4	Absent	37	Normal
				37	Absent	57	Normal
				57	Absent	64	Normal
				97	Absent	97	Thinning 10% (superior) - 15% (inferior)
		R28	F	12	Reduced	47	Normal
				17	Reduced	77	Thinning 20% (superior) - 50% (inferior)
				41	Reduced		
				76	Absent		
		R42	M	3	Normal	33	Normal
				11	Normal	62	Normal
				27	Normal		
				60	Normal		
		R46	F	3	Absent	34	Normal
				11	Absent	62	Normal
				27	Absent		
				60	Absent		
		R48	M	5	Reduced	26	Normal
				8	Reduced	61	Normal
				25	Reduced		
				60	Reduced		
		R50	F	5	Normal	26	Normal
				27	Normal	61	Normal
				60	Normal		
		R63	M	2	Absent	-	-
				9	Absent		
				29	Absent		
		R71	M	6	Reduced	6	Normal
				8	Reduced	13	Normal
				15	Reduced		

b. Study animals - OCT not performed

RPGRIP1	*MAP9*	Dog ID	Sex	Age (m)	Cone amplitude
+/+	+/del	R7	F	4	Normal
+/ins	+/+	R36	M	7	Normal
	+/del	R4	F	4	Normal
		R6	F	4	Normal
		R8	F	4	Normal
		R12	M	38	Normal
		R13	F	38	Normal
		R16	M	5	Normal
		R41	M	7	Normal
		R43	M	7	Normal
	del/del	R22	F	5	Normal
		R26	M	12	Normal
				16	Normal
ins/ins	+/+	R47	M	5	Normal
		R49	M	5	Absent
				8	Absent
		R51	F	5	Normal
		R53	M	5	Normal
		R54	F	5	Normal
		R57	F	5	Absent
				7	Absent
		R58	F	5	Absent
				7	Absent
		R60	M	1.4	Absent
				33	Absent
		R61	M	2	Absent
		R74	F	7	Absent
		R77	F	2	Absent
		R79	F	2	Normal
		R80	F	2	Normal
	+/del	R29	F	12	Absent
				15	Absent
		R40	M	3	Normal

		R72	M	7	Reduced	15	Normal
				15	Reduced		
	del/del	R62	M	2	Absent	9	Normal
				4	Absent	17	Normal
				9	Absent	19	Thinning 10% (superior) - 20% (inferior)
				14	Absent	30	Thinning 20% (superior) - 50% (inferior)
				19	Absent		
				29	Absent		
		R67	F	2	Absent	9	Normal
				3	Absent	17	Thinning 5% (superior) - 10% (inferior)
				9	Absent	19	Thinning 5% (superior) - 15% (inferior)
				14	Absent	30	Thinning 5% (superior) - 20% (inferior)
				19	Absent		
				29	Reduced		
		R70	M	6	Absent	7	Normal
				8	Absent	13	Normal
				13	Absent	17	Thinning 5% (superior) - 10% (inferior)
				17	Absent		

		R44	M	3	Normal
		R52	M	5	Reduced
		R55	F	5	Absent
				7	Absent
		R56	F	5	Absent
				7	Absent
		R64	M	2	Absent
		R66	M	2	Reduced
		R68	M	6	Normal
		R69	M	6	Absent
		R73	M	7	Absent
		R75	F	7	Reduced
		R76	M	2	Absent
	del/del	R18	M	5	Absent
		R21	F	5	Absent
		R78	F	2	Absent

Supplemental Table 4. Summary of IHC results

Antibody	Wild type	*RPGRIP1*$^{+/ins}$*MAP9*$^{+/+}$	*RPGRIP1*$^{ins/ins}$ *MAP9*$^{+/+}$ (normal-cone ERG)	*RPGRIP1*$^{ins/ins}$ *MAP9*$^{+/+}$ (n=2); *MAP9*$^{+/d}$ (n=1) (absent-cone ERG)	Co-localization L/M-opsin	Co-localization S-opsin
Rho	Normal Rod photoreceptor structure				No	No
hCAR	Normal cone OS, IS and pedicle			Diminutive cone structure, shorter cone OS and IS	Yes	Yes
L/M opsin	Localized in L/M-cone OS			Localized in L/M-cone OS with distinct mislocalization to cone IS and pedicle	n/a	No
S opsin	Localized in S-cone OS			Localized in S-cone OS with mislocalization to cone inner segment and pedicle	No	n/a
GαT2	Localized in cone OS			Reduced labelling of cone OS. Few short and narrow cone OS	Yes	Yes
GγT2	Localized in cone OS		Localized in OS and labelled IS less intensely	Weak labelling of cone OS	Yes	Yes
GRK7	Localized in cone OS			Faintly labelled cone OS	Yes	Yes
CNGA3	Localized in cone OS			Very little labelling in cone OS	Yes	Yes
CNGB3	Localized in cone OS			Very weak labelling of S-cone OS but no labelling of L/M-cone	No	Yes
GNB3	Labelled cones and very faintly bipolar cells			Faintly labelled cones and bipolar cells	n/a	n/a
PNA	Labels cone matrix sheath				n/a	n/a
RGS9	Labelled OS of rods and cones			Labelled the OS of rods and cones (faintly)	Yes	Yes
GNB5	Labelled OS of rods and cones			Labelled the OS of rods and cones (faintly)	Yes	Yes
R9AP	Labelled OS of rods and cones			Labelled the OS of rods and cones (faintly)	Yes	Yes
GC1	Labelled OS of rods and cones			Labelled rods but failed to label cone OS	No	No
Acetylated α tubulin	Labelled axoneme and transition zone of PSC in both rods and cones			Faintly labelled axoneme and transition zone of PSC in both rods and cones; significantly shorter cone axoneme and transition zone	Yes	Yes
Rootletin	Labelled both rod and cone ciliary rootlet of PSC			Faintly labelled both rod and cone ciliary rootlet of PSC	Yes	Yes
MAP9	Labelled both rod and cone axoneme of PSC			Labelled both rod and cone axoneme of PSC but with low intensity; significantly shorter cone axoneme	Yes	Yes
Synaptophysin	Punctate synaptic boutons in OPL and IPL				n/a	n/a
CtBP2	Horseshoe-like ribbon structures in OPL				n/a	n/a
SNAP25	Punctate synaptic boutons in OPL and IPL				n/a	n/a
Goα	Labelled ON-Bipolar cells in OPL and IPL with no dendritic retraction				n/a	n/a
PKCα	Labelled rod-Bipolar cells in OPL and IPL without any rod dendritic retraction				n/a	n/a
GFAP	No activation of Müller cells				n/a	n/a

IPL, inner plexiform layer; IS, inner segment; OPL, outer plexiform layer; OS, outer segment; PSC, photoreceptor sensory cilium; n/a, not applicable.

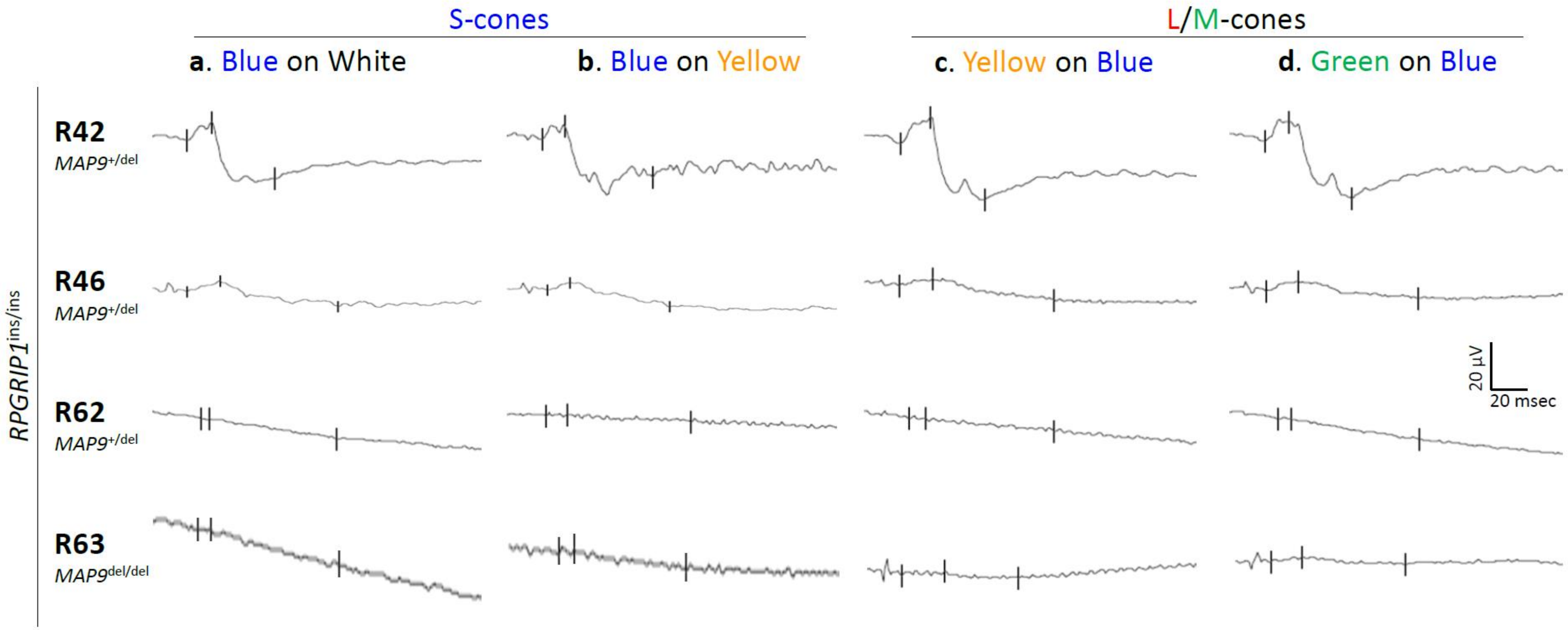

Supplemental Figure s1. S- and L/M-cone responses are equally affected in reduced- or absent-cone ERG retinas.

S-cone function was assessed by a blue LED flash on a white (a) or yellow (b) background, and L/M-cone by a yellow (c) or green (d) LED flash on a blue background. *RPGRIP1*$^{ins/ins}$ *MAP9*$^{+/del}$ animals that are normal (R42), cone ERG$^{absent\ (-severely\ reduced)}$ (R46), and cone ERG^{absent} (R62) as well as a double homozygous mutant with cone ERG^{absent} (R63) was tested. There was no selective rescue or reduction in S- or L/M-cone function, and both cone types were equally affected.

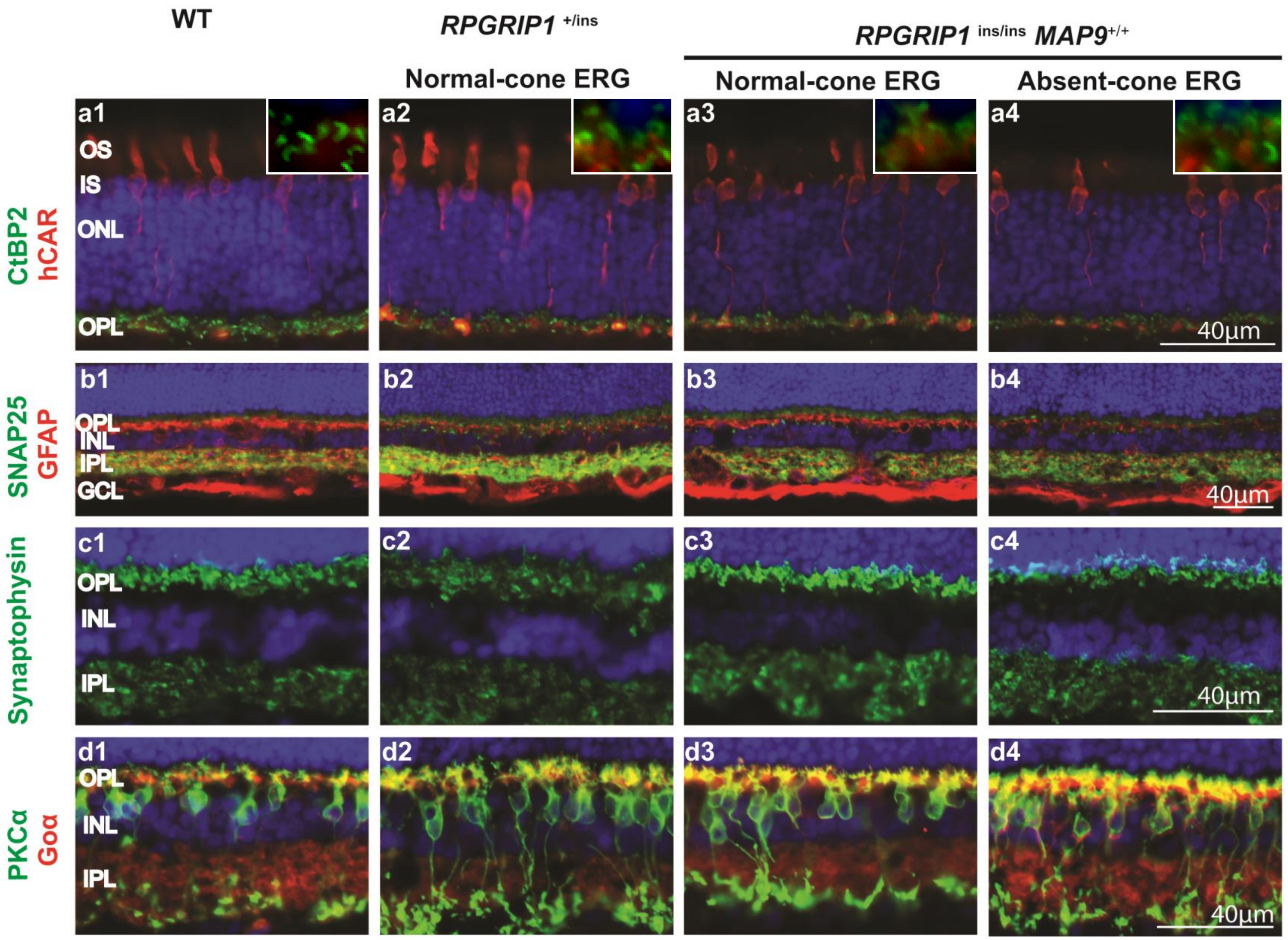

Supplemental Figure s2. Morphologically normal synaptic layers, bipolar and Müller cells as observed by fluorescence microscopy. Labelling with antibodies against synaptic terminal proteins **a**) CtBP2, **b**) SNAP25, and **c**) Synaptophysin in retinas from **1**) WT, **2**) *RPGRIP1*$^{+/ins}$, and **3**) normal- or **4**) absent-cone ERG *RPGRIP1*$^{ins/ins}$ mutants showed comparable number of puncta in the OPL and IPL. CtBP2 labelling showed comparable labelling of the horse shoe-like ribbon structures in the OPL as in the magnified image shown as insert (**a1-4**). No GFAP activation of Müller cells was observed (**b1-4**). Goα and PKCα labelling showed comparable composition of bipolar cells across different samples with no sign of axonal or dendritic retraction (**d1-4**).

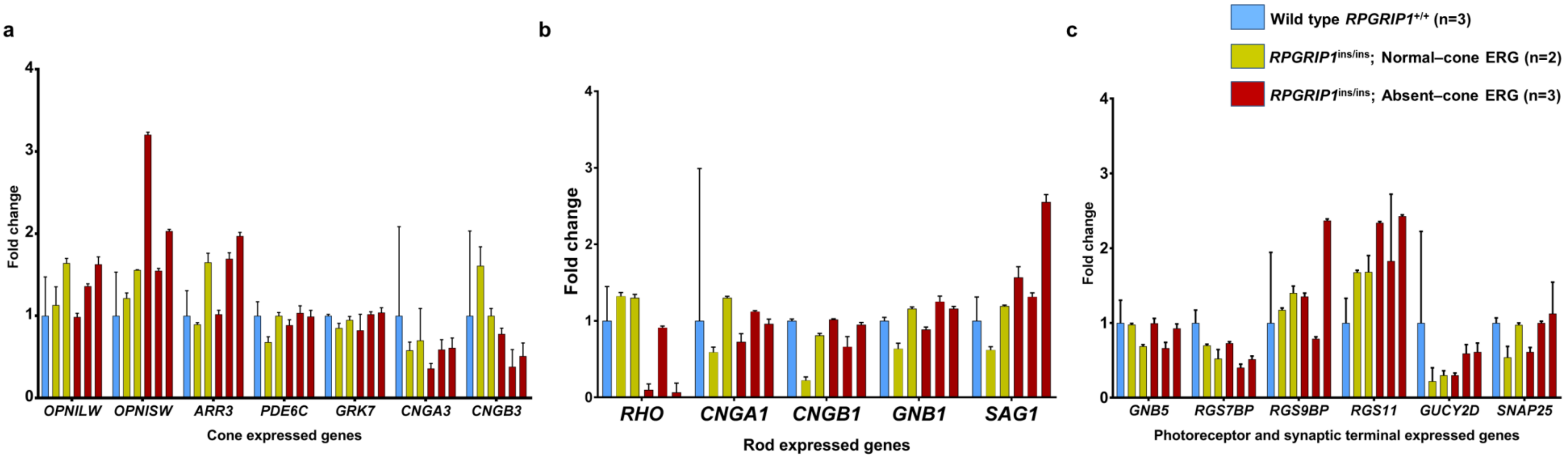

Supplemental Figure s3. Comparable transcription of selected genes in wild type and mutants.

Selected genes expressed in **a**) rods, **b**) cones, **c**) both photoreceptor types and synaptic terminal do not show any significant difference between wild type (n=3), normal- (n=2) and absent- (n=3) cone ERG *RPGRIP1*$^{ins/ins}$ retinas. Due to limited tissue/RNA availability from the cone ERG^{absent} samples, qRT-PCR experiments were performed on any given 3 of the 6 cone ERG^{absent} mutant indicated with a blue asterisk in Figure 1. Due to variation within the groups, each animal in the *RPGRIP1*$^{ins/ins}$ mutant groups are shown as individual bars. Error bars represent SE.

Supplemental Video s1. Comparable vision-guided navigation between normal- and absent-cone ERG *RPGRIP1*$^{ins/ins}$ animals.

a: A normal-cone ERG dog (R42; *RPGRIP1*$^{ins/ins}$ *MAP9*$^{+/del}$) navigates through an obstacle course without hesitation or collision.

b: An absent-cone ERG dog (R46; *RPGRIP1*$^{ins/ins}$ *MAP9*$^{+/del}$) that is a littermate of the dog shown in (a) also navigates through an obstacle course without hesitation or collision.

c: Side-by-side comparison of the video in (a) and (b) demonstrating comparable speed and precision of navigation between the two littermate dogs that have strikingly different cone ERG responses.
